# Regulation of RPE65 expression in human retinal pigment epithelium cells

**DOI:** 10.1038/s41598-025-12926-3

**Published:** 2025-07-25

**Authors:** Olga A. Postnikova, Samuel William, Sheetal Uppal, Steven L. Bernstein, Eugenia Poliakov, Igor B. Rogozin, T. Michael Redmond

**Affiliations:** 1 Laboratory of Retinal Cell & Molecular Biology, National Eye Institute, NIH, Bethesda, MD 20892 USA; 2 Departments of Ophthalmology and Visual Sciences, and Anatomy and Neurobiology, School of Medicine, University of Maryland, Baltimore, MD 21201 USA; 3 National Center for Biotechnology Information, National Library of Medicine, National Institutes of Health, Bethesda, MD 20892 USA; 4 Present Address: USDA-ARS, NEA, BARC, Animal Biosciences and Biotechnology Laboratory, Beltsville, MD 20705 USA; 5 Present Address: Life Science Research Centre, Faculty of Science, University of Ostrava, Ostrava, 710 00 Czech Republic

**Keywords:** Retinal pigment epithelium, Retina, RPE65, Transcription, Translation, Ribosome, Nicotinamide, Pyruvate, MicroRNAs, Biochemistry, Molecular biology

## Abstract

The visual cycle is an important pathway in the retinal pigment epithelium (RPE) which regenerates 11-*cis* retinal chromophore for the retinal photoreceptors. The central enzyme in the visual cycle is RPE65 retinol isomerase. Expression of RPE65 mRNA and protein levels are significantly lower in RPE cell culture models when compared to native RPE. This limits the use of these models to study the visual cycle. To determine the main drivers of RPE65 regulation we compared the transcriptional profiles of native and cell culture models of RPE with various levels of RPE65 expression. We also compared the levels of RPE65 expression between ARPE-19 cells grown in media supplemented with 1 mM pyruvate (PYR) or 10 mM nicotinamide (NAM). In addition, we performed experiments directed at transcriptional and translational regulation of RPE65. We show that RPE65 mRNA and protein expression is significantly higher in NAM media grown cells than PYR cells. Transfection of cells with a variety of different vectors containing RPE65 ORFs with different promoters, codon optimization, IRES, 3’ UTRs, suggest that translational effects are less important than transcriptional status. Importantly, we found that feeding with rod outer segments (ROS) decreases RPE65 expression in NAM grown cells, suggesting that certain primary functions of the RPE (here, visual cycle and phagocytosis) are not positively linked. Analysis of differentially regulated microRNAs (miRs) provides a basis for this downregulation. It appears that the regulation of RPE65 expression in ARPE-19 cells, in particular, is multifactorial, involving primarily metabolic and transcriptional status of the cells, with translation of RPE65 mRNA playing a smaller role.

**Supplementary Information:**

The online version contains supplementary material available at 10.1038/s41598-025-12926-3.

## Introduction

The retinal pigment epithelium (RPE) is a monolayer of pigmented cells located between the choroid and retinal photoreceptors. Maintenance of the retina and of vision is highly dependent on RPE metabolic processes, trafficking of metabolites, etc. Mammalian vision depends critically on constant production of 11-*cis* retinal visual chromophore by the visual cycle, a retina/RPE-specific metabolic pathway. RPE65 retinol isomerase, the central component of the visual cycle, catalyzes the rate-limiting conversion of all-*trans* retinyl esters into 11-*cis* retinol^1–3^ which is further oxidized enzymatically to 11-*cis* retinal. Mutations in the *RPE65* gene lead to a form of Leber congenital amaurosis (LCA), an inherited retinal degeneration. However, treatment of *RPE65*-associated LCA is now possible with adeno-associated virus-mediated gene augmentation^4^. While this leads to partial recovery of vision in treated patients, RPE65 expression is not completely restored.

While RPE65 mRNA and protein are both very abundant in native RPE, explanted bovine RPE rapidly lose RPE65 protein expression after 2 weeks in primary culture but retain RPE65 mRNA for up to 7 weeks with no immunodetectable RPE65 protein^5^. It was earlier proposed that RPE65 mRNA 3′-untranslated region (UTR) contains elements key to translational regulation^6^. In the past 10 years there has been increased interest in the differentiation of RPE from induced pluripotent stem cells (iPSCs) and embryonic stem cells (ESCs) for transplantation or as models of disease (“disease in a dish”). However, a common feature of these models is low expression of visual cycle genes, and this is especially true for RPE65 gene expression. Important in this regard is a report^7^ that lentiviral mediated RPE65 gene transfer in human iPSC-derived RPE cells markedly increased RPE65 mRNA, but only modestly the expression of RPE65 protein.

ARPE-19 is a widely used human RPE cell line introduced in 1996^8^. Since 2010, > 100 studies have been published each year utilizing ARPE-19 cells. Many such studies use ARPE-19 cells in an undifferentiated pre-confluent state to allow for transfection or for a shorter experimental timeframe, but their expression of RPE-associated genes generally is low or absent. In contrast, we and others have found^9,10^ that, with appropriate differentiation, ARPE-19 cells attain a phenotype and gene expression profile similar to native RPE. However, though RPE65 mRNA is expressed at low levels in these cells, there is no translation of RPE65, suggesting a mechanism in cultured cells inhibiting both RPE65 transcription and translation. Even up to the present time, the question as to how RPE65 expression is regulated in RPE cells is still not well resolved.

Masuda et al.^11^ reported that the transcription factor (TF) SOX9 acts synergistically with another TF OTX2 to activate RPE65 and RLBP1 transcription, while ChIP experiments in human fetal RPE showed that SOX9 and OTX2 bind to the RPE65 promoter. Pyakurel et al.^12^ suggested regulation of RPE65 gene expression by an ERK1/2 pathway, and using luciferase assays and EMSA experiments in ARPE-19 and HEK293 cells showed the binding of C-FOS and FRA-1 complexes to the AP-1 site in the RPE65 promoter region. In addition, visual cycle genes appear to be under circadian regulation with light-dependent regulation of the genes responsible for retinoid recycling (*Lrat*, *Rpe65*, and *Rdh5*) in RPE^13^. Splicing represents another level of posttranscriptional control of gene expression. Previously, we showed frequent exon skipping events involving exons 2, 3, and 7 in RPE65 mRNA. The frequency of these events was much higher in cultured RPE cell models compared to native RPE^14^. Furthermore, rates of translational initiation, elongation and ribosome recycling greatly affect protein production.

Clearly, the precise mechanism of RPE65 expression regulation remains to be elucidated fully and requires further effort to resolve. Here we compare transcriptional changes, ribosomal profile and metabolic status of ARPE-19 cells with relatively high and low levels of RPE65 mRNA in different differentiation protocols using different media. In addition, we studied the influence of 3’ UTR, IRES and codon optimization, use of different promoters, and ROS uptake on RPE65 mRNA expression and translation.

## Results

### Low expression level of RPE65 and other RPE marker genes in cell model systems compared to native tissue

To assess how similar existing cellular models of RPE (differentiated stem cells^15^ or ARPE-19 cell cultures^10^ are to native human tissue, we compared their global gene expression profile. To do so RNA-Seq data from nasal, temporal, and macular regions of human RPE/choroid were used^16^. We also included in this analysis bovine native RPE and bovine primary RPE cell culture^17^ to estimate species specific differences and the effects of culturing. Interestingly, native bovine RPE tissue did not show a very strong correlation with native human RPE (*R* ~ 0.5–0.67) (Supplementary Figure S1).

**Fig. 1:**
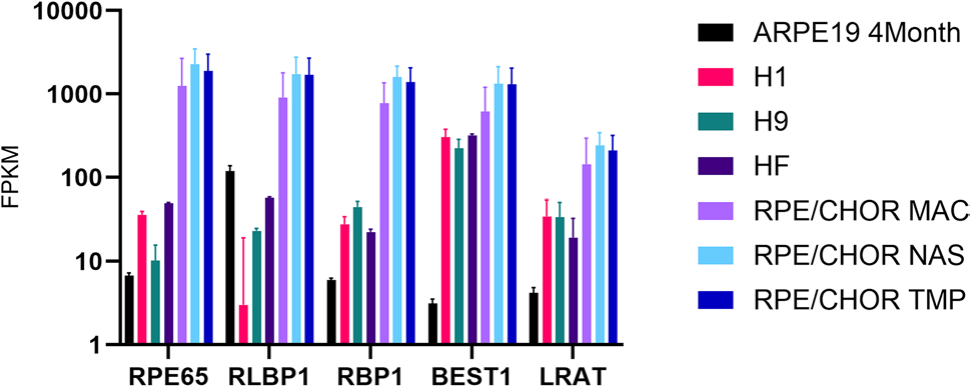
FPKM normalized expression levels of visual cycle genes from RNA-seq data. RNA-seq data was obtained from: ARPE-19 4 Month, ARPE-19 cells differentiated for 4 months under PYR protocol; H1, RPE differentiated from H1 ESC; H9, RPE differentiated from H9 ESC; HF, Human Fetal RPE cell lines; RPE/CHOR, RPE from macular (MAC), nasal (NAS), and temporal (TMP) regions of human RPE/choroid, respectively. Data was collected from (10, 15, 16).

All human-based models show more similarity between each other (R > = 0.54) and showed moderate similarity to the native RPE/choroid tissue with Pearson correlation coefficients ranging between 0.36 and 0.6. However, calculated Spearman correlation coefficients were much higher than Pearson coefficients for all comparisons (Supplementary Figure S2). Because Spearman coefficients are computed on ranks, higher S values might suggest that expression ratios between genes are quite similar between all the models and native tissue. However, the mRNA levels for many genes in the cell cultures do not reach the absolute FPKM values observed in the native tissue as illustrated in Fig. 1 for visual cycle genes. For example, RPE65 mRNA and protein are highly expressed in native RPE cells, while primary RPE cultures rapidly lose RPE65 protein first and then RPE65 mRNA^5^.

RPE differentiated from iPSCs, embryonic H1 or H9 RPE, as well as ARPE-19 cells have much lower RPE65 mRNA expression and very low protein expression that often cannot be detected by immunoblot (Supplementary Figure S3A). This phenomenon of low/no RPE65 protein expression in these model systems is not well understood.

### Increased expression of RPE65 mRNA and protein in ARPE-19 cells grown in low glucose media with nicotinamide

We used 2 protocols to differentiate ARPE-19 cells: the PYR protocol^10,18^and the NAM protocol^9^ (see Methods). After 1-month growth on laminin-coated plastic in PYR-supplemented DMEM + 1% FBS, ARPE-19 cells become pigmented and form tight junctions. Also, at this time point, ARPE-19 express many RPE marker proteins such as Bestrophin, MERTK, LRAT and others, similar to RPE differentiated from iPSC (Supplementary Figure S3A). However, RPE65 protein cannot be detected by immunoblot in the RPE-like cells^10^.

**Fig. 2:**
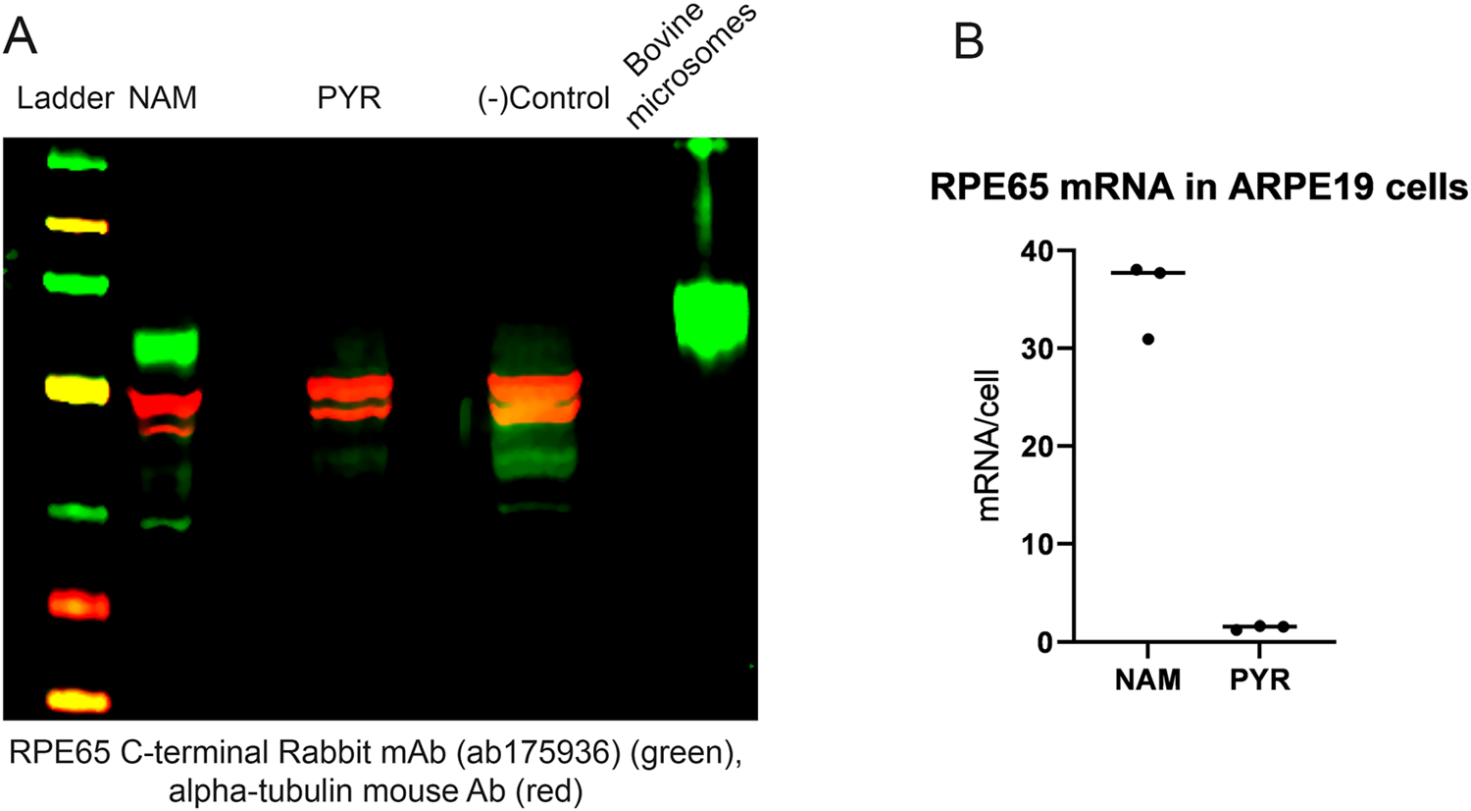
Detection of RPE65 protein and mRNA expression in ARPE-19 cultured in NAM media. **A**. Detection of RPE65 protein with rabbit monoclonal RPE65-[EPR7024(N)]-C-terminal antibody (Abcam, Cat. No. ab175936). Bovine RPE microsomes were used as a positive control. Blots were visualized using a LI-COR Odyssey CLx Imaging System. **B**. qPCR quantification of RPE65 mRNA in ARPE-19 cells differentiated under NAM or PYR protocol.

We adopted the RPE differentiation protocol of Hazim et al.^9^. Firstly, cells were differentiated using DMEM supplemented with sodium pyruvate and 1% FBS for one week and then switched to MEM-alpha supplemented with 10 mM nicotinamide (NAM)^9^. Using this protocol, RPE65 protein was detected from extracts of these cells on western blot with a custom in-house monoclonal antibody (data not shown), but when we probed RPE65 immunoreactivity with a rabbit monoclonal antibody (Abcam) a stronger signal was observed compared to the former (Fig. 2A). Immunoprecipitation experiments using the Abcam antibody were conducted on these NAM-treated ARPE-19 cell extracts to confirm specificity to RPE65 protein; bovine RPE microsomes were used as a positive control. Analysis by LC-MS confirmed the presence of RPE65-derived peptides in the NAM-cultured ARPE-19 sample. (Supplementary Table S1).

Next, we assessed quantitatively the amount of transcribed mRNA in these ARPE-19 cells. We observed a 50-fold difference in RPE65 mRNA level between the two culture protocols. There were on average 35 molecules of RPE65 mRNA per cell in NAM media-differentiated ARPE-19 cells versus 0.7 molecules per cell in the PYR/high glucose media differentiated ARPE-19 cells (Fig. 2B).

### Transcriptional changes in ARPE-19 cells under different culturing conditions

We performed RNA-seq to compare the expression profile of ARPE-19 cells differentiated under either NAM medium or PYR medium protocols using both Illumina and Nanopore sequencing platforms. Illumina RNA-seq produced about 5 times more differentially expressed genes compared to Nanopore long read sequencing (FDR < 0.01; Supplementary Table S2) We confirmed genes found to be differentially expressed by one or both methods using qPCR (Supplementary Table S3, Supplementary Figure S3B). The correlation between qPCR and Illumina log2 fold change was 0.98.

**Fig. 3:**
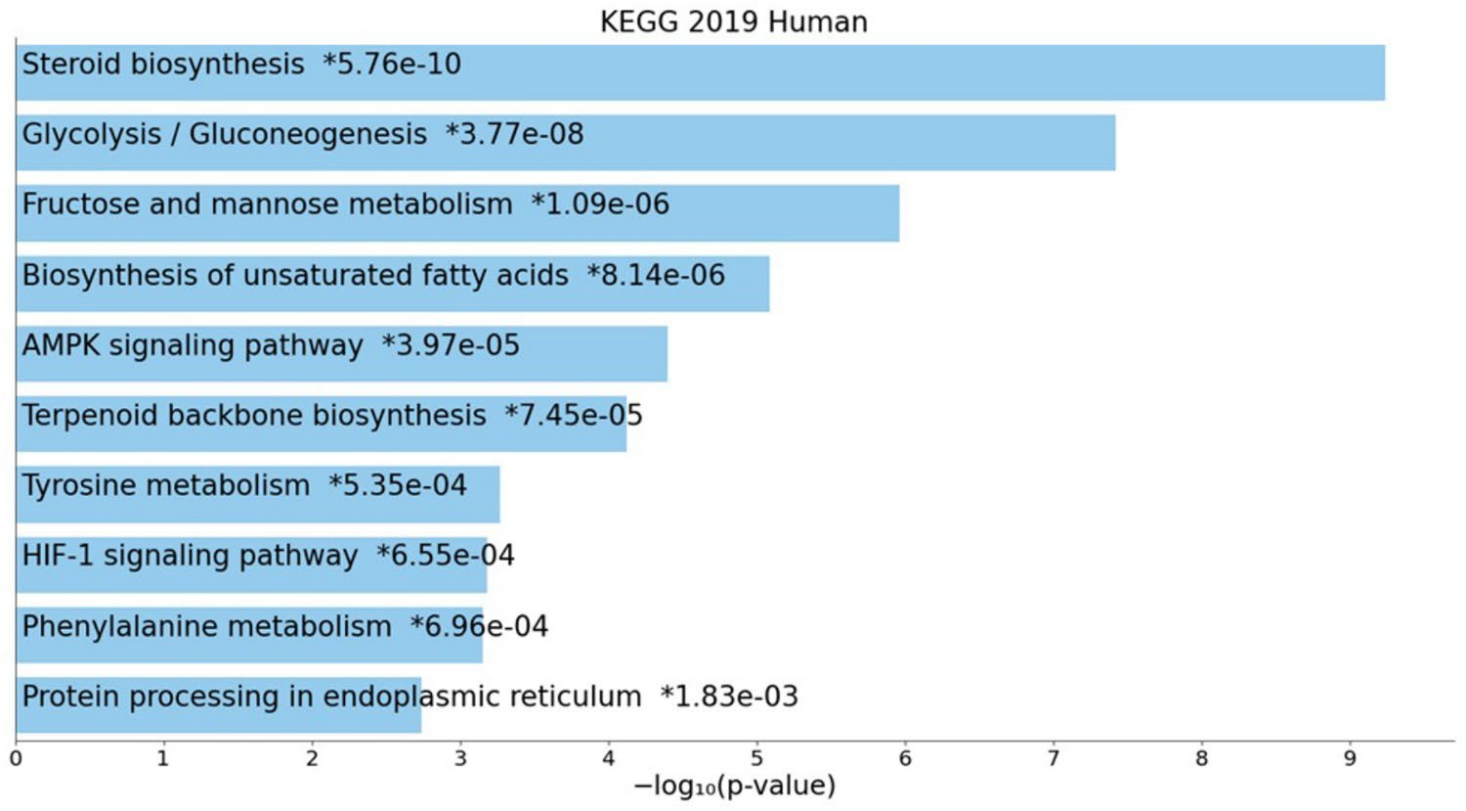
Overrepresented pathways in genes upregulated in NAM grown ARPE-19 cells compared to those grown in PYR. KEGG 2019 pathways overrepresented in ARPE-19 cells grown in NAM protocol compared to those grown in PYR media^19,20^.

In both data sets one of the most overrepresented pathways is steroid biosynthesis (Fig. 3). There was upregulation of almost all genes coding for cholesterol biosynthetic enzymes in cells grown in NAM media. We noticed activation of many corticosteroid-regulated genes, including *CXCR4*, *EPHA8*, *FKBP5*, *HVCN1*, *PER1*, *PTGER2*, *SCNN1A*, *SERPINA3*, and *TMEM72*, genes similarly upregulated in human iPSC-derived RPE^21^. We also saw downregulation of *OMG*, a gene also found to be downregulated by corticosteroids (aldosterone, cortisol, or cortisol + RU-486) in human iPSC-derived RPE^21^. Under the NAM protocol ARPE-19 cells showed increased expression of glycolysis/gluconeogenesis genes (including PFKP, *ALDOC*,* PGK1*,* GAPDH*, and *ENO1*). Other components of MEM-alpha media such as hydrocortisone increase EGF and proliferation and decrease stress-regulated genes. Taurine, an added supplement in NAM, which plays roles as an antioxidant, is implicated in osmoregulation and in suppressing EMT and oxidative stress. A reduction in oxidative stress is also evident by changes in the TGF-beta signaling pathway. Also, we noticed that cells differentiated on MEM-alpha + NAM have reduced melanin pigmentation compared to ARPE-19 grown in PYR media. This correlates with the finding that *TYR*,* TYRP1* and *DCT* were downregulated during growth in NAM media compared to PYR media (Supplementary Figure S4). Thyroid hormone, another supplement, may decrease phagocytosis through regulation of Na, K-ATPase. Also, there is a correlation between thyroid hormone and LDL cholesterol biosynthesis (Supplementary Figure S5)^22^. In addition, TTR is a top gene contributor to differences between the 2 growth conditions along with PER1 (Period Circadian Regulator 1) and SERPINA3 (Serine Proteinase Inhibitor A3). Interestingly, in our IP pulldown experiment, SERPINA3 peptides were found to be associated with RPE65 protein (data not shown).

We looked for known transcriptional regulators of RPE65 such as SOX9 and OTX2^11^, but none were differentially expressed at least ±2-fold. Interestingly, we found that members of the FOS family: FOSB and FOSL2 (part of the AP-1 transcription complex) were expressed higher under NAM treatment, 5 and 2.6-fold respectively (Supplementary Table S2). It is possible that there are other TF(s) that regulate activation of RPE65 transcription that are differentially regulated under the NAM differentiation protocol.

### Metabolic status of ARPE-19 cells grown in different media

**Fig. 4:**
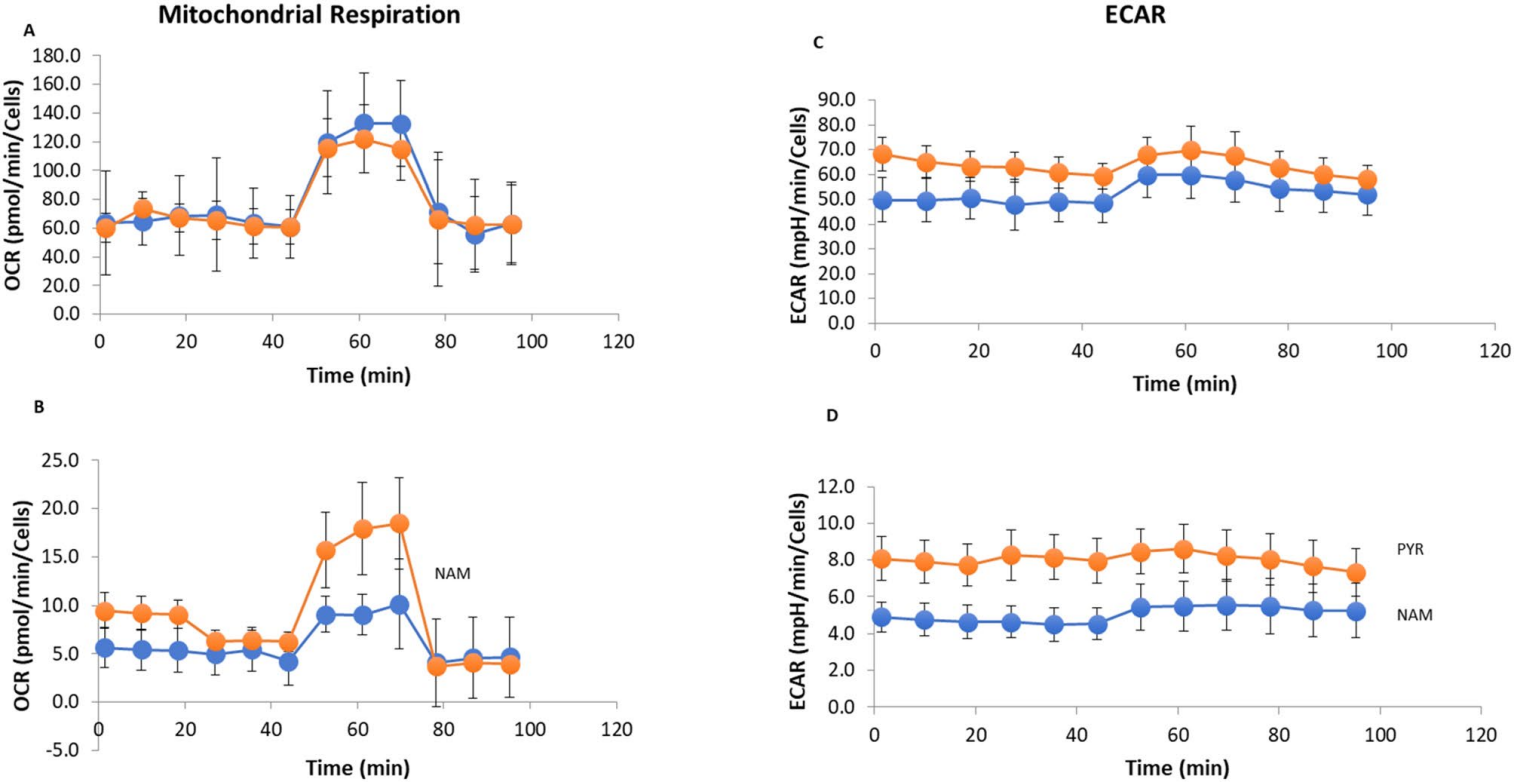
Metabolic status of ARPE-19 cells grown in NAM and PYR media. We assessed the effect of NAM and PYR media on glycolytic and mitochondrial metabolism in ARPE-19 cells. **A**, **B**, Oxygen consumption rate (OCR) and **C**, **D**, Extracellular acidification rate (ECAR) measured by Seahorse XF cell Mito Stress assay for NAM (Blue) and PYR (Orange) protocol differentiated ARPE-19 cells. **A**, **C**, OCR and ECAR assays were done in the same base media (10 mM glucose, 1 mM pyruvate and 2 mM glutamine) for both NAM and PYR protocol differentiated ARPE-19 cells. **B**, **D**, OCR and ECAR assays were done in the base media modified with 10mM nicotinamide, 5 mM glucose, 1 mM pyruvate, 2 mM glutamine for cells grown on NAM media, and base media modified with higher glucose 25 mM glucose, 1 mM pyruvate, 2 mM glutamine for cells differentiated under PYR protocol.

Kang and Hwang reported that nicotinamide enhances the quality of mitochondria in human cells through activation of autophagy^23^while Hazim et al. found that it also enhanced mitochondrial metabolism in ARPE-19 cells^24^. To test the effects of long-term NAM culture on mitochondria of ARPE-19 cells we used the Seahorse XF cell Mito Stress assay on cells differentiated using the different media protocols. First, we used the same modified medium (10 mM Glucose, 1 mM pyruvate, 2 mM glutamine) during assays with ARPE-19 cells differentiated with NAM or PYR protocols. We did not see any significant differences in mitochondrial respiration. These results suggest that ARPE-19 cells adjust their metabolism very quickly regardless of which medium in which they are grown (Fig. 4A, B). Next, we modified the assay media for cells grown on NAM medium (with addition of 10 mM NAM, 5 mM Glucose, 1 mM pyruvate, 2 mM glutamine) and higher glucose for cells differentiated under PYR protocol (with addition of 25 mM Glucose, 1 mM pyruvate, 2 mM glutamine). We found that ARPE-19 cells had higher glycolysis and oxidative phosphorylation levels when grown in PYR, compared to cells grown on NAM medium. We saw an increase in the extracellular acidification rate (ECAR) in cells on PYR medium compared to NAM medium (Fig. 4C, D) correlating with a higher glycolytic rate in PYR cells. Also, mitochondrial respiration was higher in PYR cells probably because of the higher glucose concentration used in this medium. This result agrees very well with findings from Kang^23^ that NAM decreases mitochondrial respiration in fibroblast cells. This suggests decreased ROS production and healthier mitochondria in NAM-grown ARPE-19 cells.

### RPE65 expression with different vectors and promoters

To explore how translation of RPE65 mRNA is affected by sequence (with or without codon optimization), promoter and cell type, we performed a series of transfection experiments using different vectors and RPE65 mRNA sequences in a variety of mammalian cell lines. HEK293T cells successfully translate RPE65 protein from all the constructs used. COS7 cells were able to translate detectable amounts of protein only in the codon-optimized RPE65 sequence (pQEhRPE65). When ARPE-19 cells were transfected with either pVitro2 (-> hFerH promoter-> mEF1 5’ UTR-> RPE65 mRNA -> FMDV IRES) or pcDNA (-> CMV-promoter-> t7-promoter-> RPE65 mRNA-> polyA signal) constructs we found that utilization of the pVitro2 vector increased translation of RPE65. (Supplementary Figure S6).

### Influence of 3’ UTR on RPE65 translation in different cell lines

**Fig. 5:**
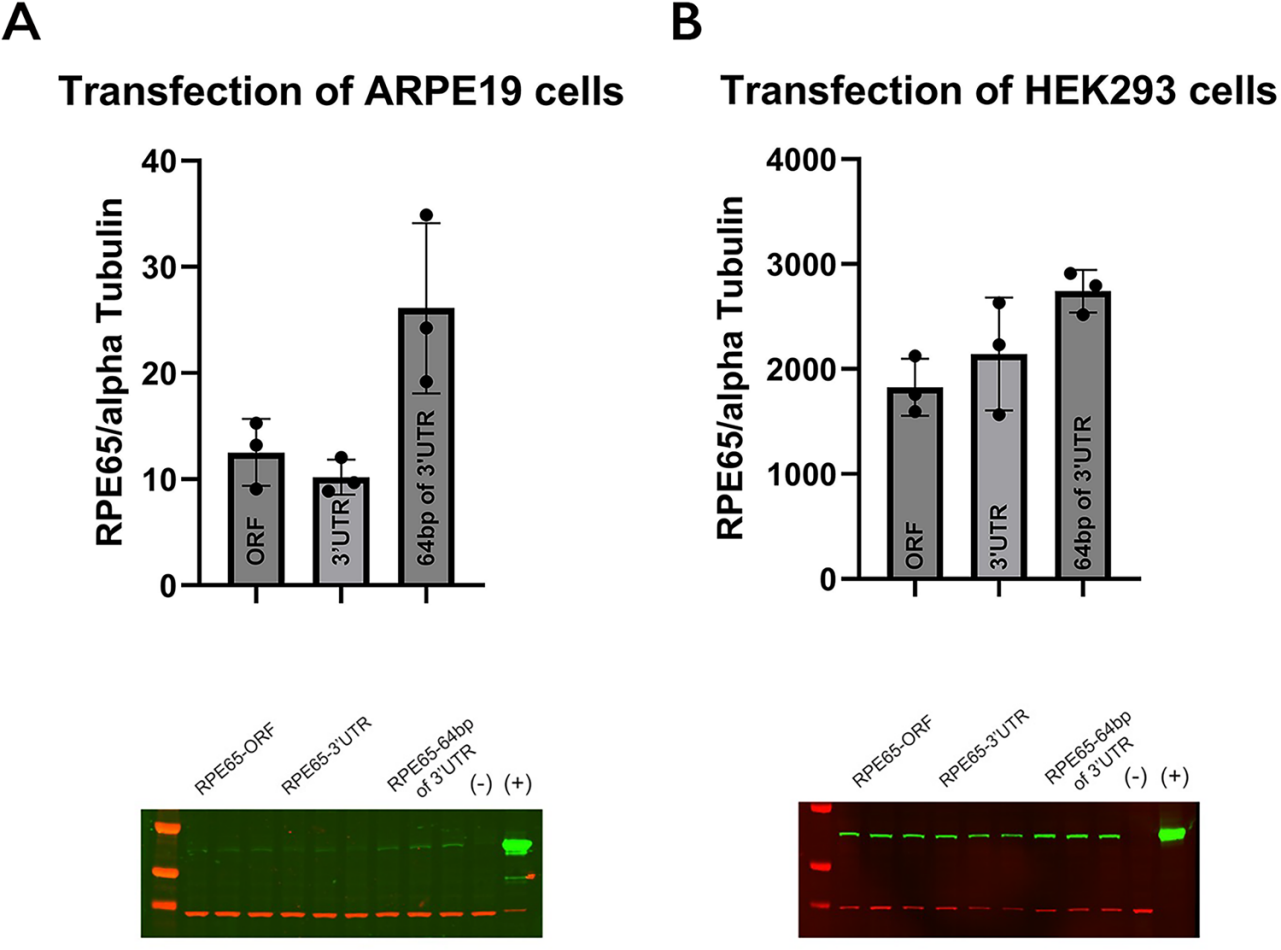
Influence of 3’ UTR length on RPE65 translation in different cell lines. RPE65 protein was quantified by immunoblot in **A**, ARPE-19 and **B**, HEK293F cells transfected with pcdna-rpe65-ORF, pcdna-rpe65- ORF − 3’UTR and pcdna-rpe65- ORF -first 64 bp of 3’UTR. All protein levels were normalized to alpha-tubulin protein levels (lower panels). (-), negative control, untransfected cells; (+), positive control, bovine RPE microsomes. The original blots are presented in Supplementary Figure S13.

Next, we asked what were the effects of 3’ UTR on translation of RPE65. Previously, it has been reported that the 3’ UTR can affect translation of RPE65 protein in in vitro transcription/translation systems^6^. We generated RPE65 constructs with different 3’ UTR lengths ranging from the ORF alone to full length 3’ UTR. We noted that when ARPE-19 cells and suspension-grown HEK293F cells were transfected with pcDNA carrying the RPE65 ORF + initial 64 bp of its 3’ UTR that there was increased protein production compared to either the ORF alone or to constructs containing the full-length 3’ UTR (Fig. 5). We observed far higher transfection and transcription efficiency in HEK293F (Fig. 5B) than in ARPE-19 (Fig. 5A), based on mRNA and protein levels.

### Use of viral transduction to enhance expression of RPE65

To determine if we could increase the efficiency of delivery of RPE65 constructs and thereby enhance RPE65 expression in ARPE-19 cells, we made VSVG spike protein-pseudotyped viral particles carrying full length RPE65 mRNA in the pLenti vector under a CMV promoter. As a positive control, we used VSVG spike protein-pseudotyped particles carrying GFP in the same pLenti vector. Using this positive control, we determined the quantity of viral particles needed for transduction of ARPE-19 cells to 80–90% GFP positivity (Supplementary Figure S7). Next, we transduced ARPE-19 cells grown on either PYR or NAM media with the same determined amount of pseudotyped viral particles (see Methods) carrying RPE65 mRNA. Relative qPCR quantification of RPE65 mRNA revealed the presence of 2 times higher RPE65 mRNA in 7-day ARPE-19 cell cultures grown on NAM media compared to cells grown in PYR media (Supplementary Figure S8A). Furthermore, the level of RPE65 mRNA was dose-dependent with respect to the number of viral particles transfected. We probed RPE65 protein expression with the Abcam rabbit monoclonal antibody and we could detect protein in both NAM and PYR grown ARPE-19 transduced cells (Supplementary Figure S8B). We also found that the amount of protein positively correlated with the amount of mRNA in ARPE-19 cells. Using our custom monoclonal antibody the limit of detection is much higher than that of the rabbit monoclonal Abcam antibody (based on our western blots probed on similar samples). These data suggest that the major regulation of RPE65 expression is due to transcriptional regulation rather than to translational regulation, as the amount of protein correlates very well with the amount of RPE65 mRNA in cells. It is still not known which TF(s) is the direct regulator of RPE65 transcription in ARPE-19 cells under NAM treatment.

### Translational efficiency by ribosome profiling

**Fig. 6:**
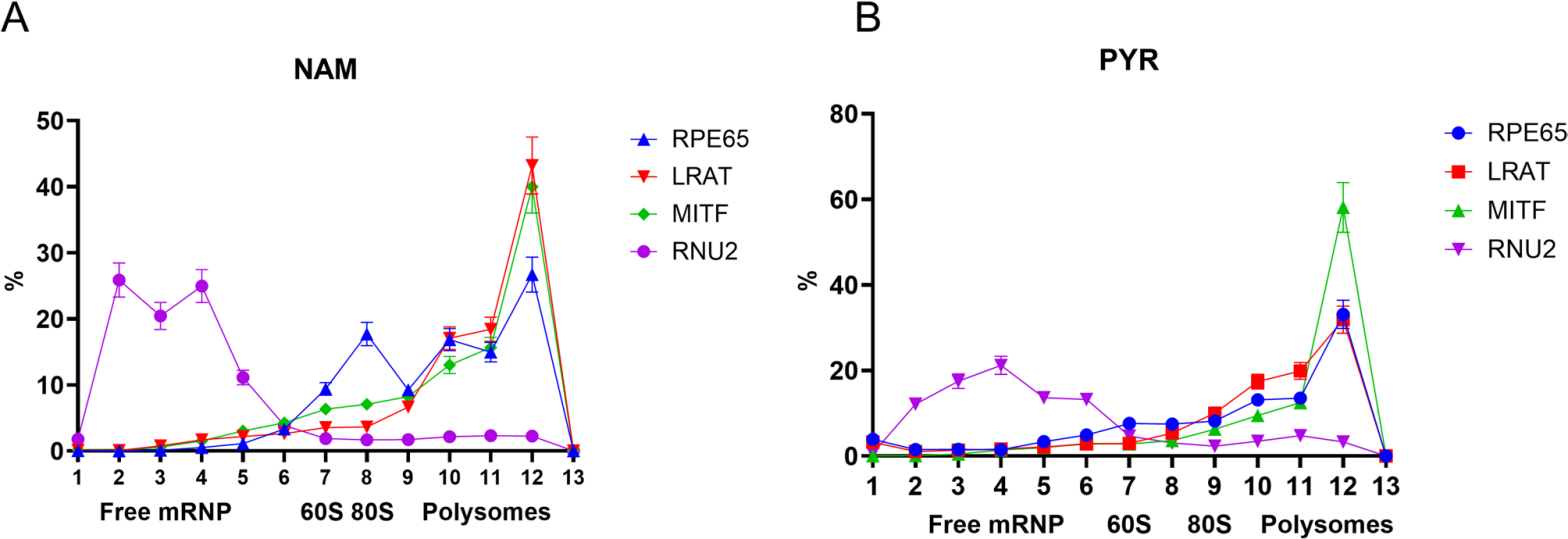
Ribosomal profiling fractionation of RPE65 mRNA in ARPE-19 cells. **A**, NAM cultured cells; **B**, PYR cultured cells. Percentage distributions of RPE65, LRAT, MITF mRNAs in each fraction during ribosomal profiling on 7–47% sucrose gradients. The protein-coding mRNAs LRAT and MITF are used as positive controls, while the non-coding, and non-translated RNA RNU2 is used as a negative control. Fractions 1–5 - free mRNP, fraction 6–7–60 S ribosomal subunit, fraction 8 - monosomes and fraction 9–12 - polysomes. RNAs were quantitated by RT-PCR.

Next, we inquired into the translational status of RPE65 mRNA. We performed 7–47% sucrose density gradient fractionation of differentiated ARPE-19 cell lysates grown in NAM or PYR media. We found that a fraction (~ 25–30%) of RPE65 mRNA is bound to ribosomes. When we compared ribosome profiling of ARPE-19 cells differentiated on NAM or PYR media, we saw that there was no significant difference in the percentage of RPE65 mRNA in polysome-bound fractions (Fig. 6). However, there was an increased percentage of RPE65 mRNA associated with the preinitiation complex fraction in the NAM media samples. This suggests possible problems during cap-dependent initiation of RPE65 mRNA translation. Additionally, when we use constructs containing an IRES element we see an increase in protein production. Most of the native RPE65 mRNA is not bound to polysomes, suggesting a low efficiency of RPE65 mRNA translation. The ratio between the polysome-bound fraction to all other fractions indicates translational efficiency, so we compared this ratio between 3 RPE-expressed genes (RPE65, LRAT and MITF). RNU2, a non-coding RNA, served as a control (Supplementary Figure S9). Fractionation was performed for already differentiated cells, and not with dividing cells that are more active in transcription and translation. The lower ratio for RPE65 mRNA relative to the other RPE genes indicates a possible inhibitory mechanism at the translational level in ARPE-19 cells (Table 1).

In addition, the RPE65 isoform with skipped exon 7 had a different profile with the maximum percentage at lighter polysomes compared to the full length RPE65 isoform, as measured with primers in exons 13–14 (3’ UTR) or 10–11 (*RPE65*’s longest intron, 6.6 kb, is located between exons 10–11). The splicing of this long intron could be a rate limiting step. Finally, it is interesting to note the high A content in RPE65’s Kozak sequence^25^compared to other visual cycle/RPE genes:

 RPE65 −201 AAAAUGU.

TYR-201 AGAAUGC.

LRAT-206 GGGAUGA.

LRAT-205 AGGAUGA.

RLBP1-201 AACAUGU.

**Table 1:** Translational efficiency of RPE-expressed genes.

NAM	RPE65	LRAT	MITF	RNU2
Free + 60 S + monosomes (%)	58.31±2.62	38.36±4.60	44.36±6.65	95.42±1.91
Polysomes (%)	41.69±1.88	61.64±7.40	55.64±8.35	5.89±0.12
Ratio	0.71	1.61	1.25	0.06

PYR	RPE65	LRAT	MITF	RNU2
Free + 60 S + monosomes (%)	53.30±6.40	48.16±7.71	29.38±8.52	91.89±4.59
Polysomes (%)	46.70±5.60	51.84±8.29	70.62±20.48	8.11±0.41
Ratio	0.88	1.08	2.40	0.09

RPE65 mRNA shows lower translational efficiency than either LRAT or MITF mRNAs. The ratio of polysome-bound to all other fractions was calculated from sucrose fractionation of mRNA of RPE genes of ARPE-19 cells expressed in either PYR or NAM. RNU2 serves as control. Data was calculated as % of mRNA quantitated by qrtPCR; *n* = 3.

However, it is not clear if this would contribute to translation initiation efficiency.

### Splicing of RPE65 gene transcripts

**Fig. 7:**
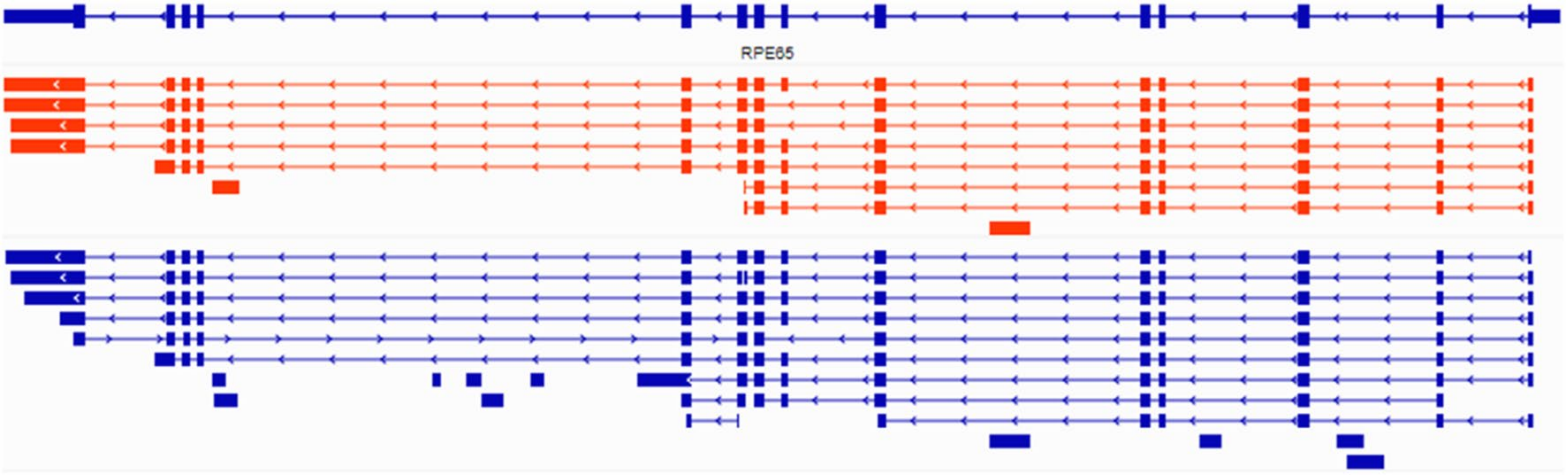
Integrative Genomics Viewer (IGV) plots of RPE65 splice isoforms. Transcript variants of the RPE65 gene were analyzed by Oxford NanoporeTech PCR based cDNA sequencing and the transcripts compared by IGV. The upper sub-panel represent the NCBI annotation of the RPE65 gene. In the middle, the red splice isoforms were assembled from mRNAs from ARPE-19 cells differentiated using the PYR protocol, while the lower blue splice isoforms were assembled from mRNAs from postmortem human RPE. Arrows indicate the direction of isoform (sense or antisense).

Splicing is another level of regulation of gene expression. To address this in RPE65, we used long read sequencing to compare RPE65 isoforms in native postmortem human RPE versus ARPE-19 cells cultured using the NAM protocol. We employed PCR-based Nanopore libraries as they produce more usable data compared to the direct RNA or cDNA sequencing approaches. Analysis of Nanopore data showed potential novel exon transcripts present in intron 5 and 10 for both ARPE-19 and native human RPE, while novel isoforms were observed in both native human RPE and ARPE-19 (Fig. 7). Some of these transcripts are common: for example, transcripts with skipping of exon 7. This isoform was also confirmed by Sanger sequencing of RPE65 clones. Quantification of this isoform by qPCR and read mapping suggest that this variant is less than 10% of RPE65 mRNA^14^. It is interesting that only in native human RPE we found an antisense RPE65 isoform, but the expression level of this isoform is very low (0.5%). Expression of RPE65 was much higher in postmortem native RPE compared to cultured cells, so some low-level isoforms may not be detected in the latter by Oxford Nanopore sequencing. In general, we do not observe any significant changes in splicing of RPE65 mRNA in ARPE-19 versus native human RPE. (Fig. 7).

### Transport from nucleus to cytoplasm

Finally, another possible step in translational control involves mRNA export from the nucleus. To address this question, we extracted RNA from nucleus and cytoplasm of differentiated ARPE-19 cells and measured the levels of RPE65 mRNA in each, using cytoplasmic GAPDH RNA and the nuclear-localized U2 spliceosomal RNA as controls. We found that most of the RPE65 mRNA was present in the cytoplasmic fraction (Supplementary Figure S10).

### Effect of rod outer segment (ROS) feeding on RPE65 expression

**Fig. 8:**
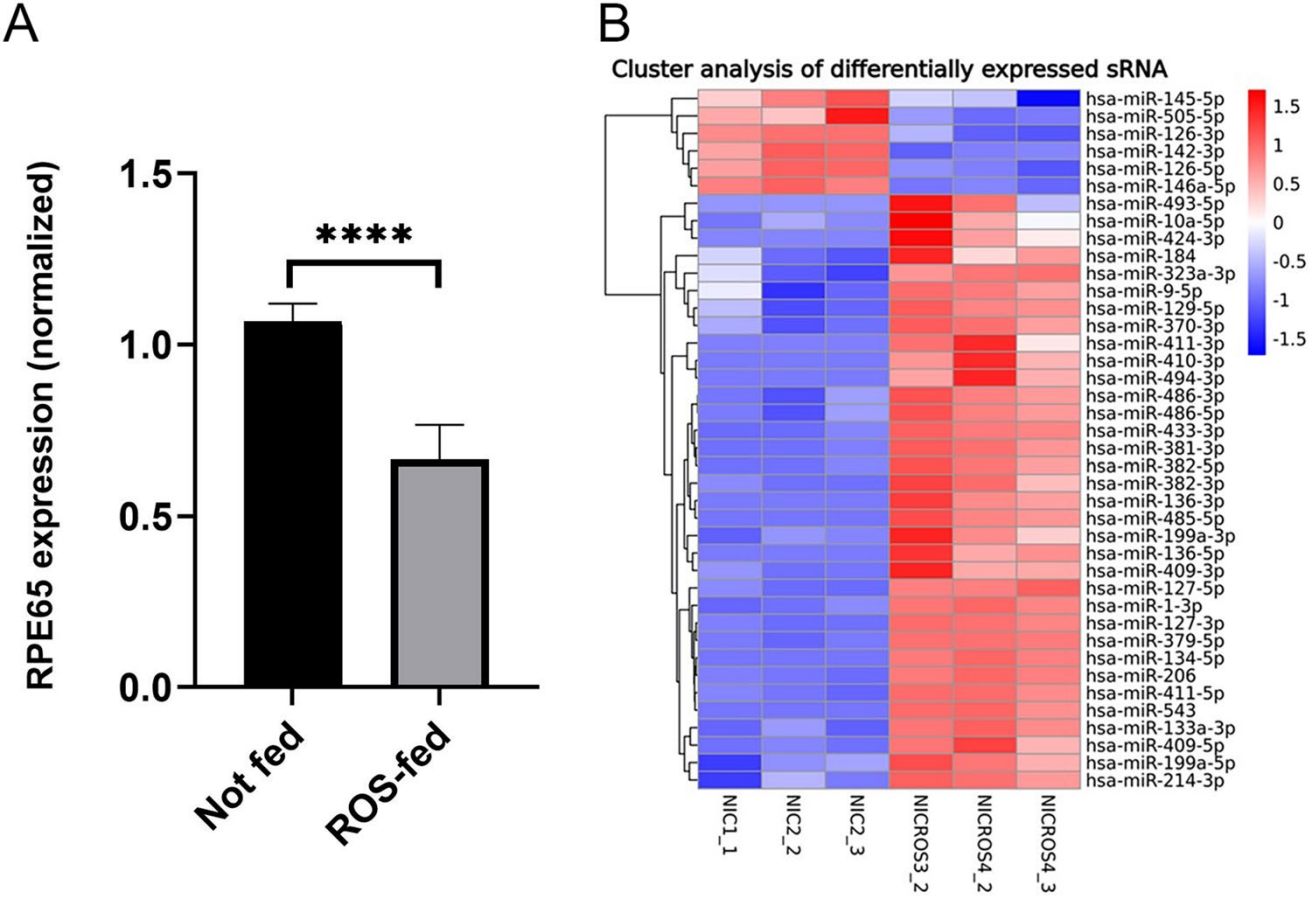
Effect of feeding ROS on NAM-cultured ARPE-19 cells. **A**. ROS feeding reduces the expression of RPE65 mRNA in ARPE-19 cells. RPE65 transcript was measured by qPCR; *n* = 3. **B**. Differentially regulated miRs in ROS-fed ARPE-19 cells. Cluster analysis heatmap of down-regulated and upregulated miRs in ROS-fed NAM-cultured ARPE-19 cells (NICROS) compared to unfed NAM- cultured ARPE-19 cells (NIC). Red represents miRNAs with higher expression levels, blue represents miRNAs with lower expression levels. Color from red to blue represents the log10(TPM + 1) value from larger to smaller; ****=*p* < 0.0001, *n* = 3.

To test the hypothesis that engaging a core physiological function of RPE cells could increase the expression of visual cycle genes, we fed ROS to NAM-cultured ARPE-19 cells. However, we found that this had a negative impact on RPE65 expression in NAM-differentiated ARPE-19 after 24-hours (Fig. 8A). The amount of RPE65 mRNA was significantly decreased in NAM-differentiated ARPE-19. This was a surprising result. To investigate this further we examined changes in microRNA (miR) expression in these cells. We found that there were 17 miRNAs expressed only without ROS and 41 expressed only with ROS feeding. Analysis of differentially expressed miRNA in NAM grown ARPE-19 revealed 34 up-regulated miRNAs after ROS treatment and 6 down-regulated, at significance levels < 0.05 (Fig. 8B).

Four differentially expressed miRNAs that are predicted to interact with human RPE65^26^ are upregulated (miR-129-5p, miR-214-3p, miR-410-39, and miR-9-5p), while one is downregulated (miR-145-5p). Collectively, these may explain partially the decrease in RPE65 mRNA expression. MiR-126-3p, highly expressed in NAM ARPE-19 and involved in maintenance of epithelial properties^27^is downregulated more than 4-fold under ROS treatment. Hsa-miR-146a-5p expression decreased in cells treated with ROS. We previously found that miR-146a-5p expression in RPE is highly induced by proinflammatory cytokines, potentially via the JAK/STAT pathway^28^. It negatively regulates inflammatory processes due to its ability to suppress the NF-κB signaling pathway by targeting IRAK1 and TRAF6 for translational repression^29^. Hsa-miR-206, which may be involved in regulating autophagy, is highly upregulated by ROS feeding. Autophagic malfunction includes the decreased expressions of autophagy-related proteins, such as AMP-activated protein kinase (AMPK), sirtuin 1 (SIRT1), and autophagy-related protein 8a (ATG8a)^30–32^. Interestingly, overexpression of miR-206 downregulates OTX2 and SOX9 and other TFs. When analyzed by qPCR these genes were modestly reduced (Supplementary Figure S11). OTX2 and SOX9 are known to bind promoter regions of visual cycle genes^33^^11^. FOSL2 and MITF were also downregulated but FOSB was not seen (Supplementary Figure S11). This would provide a further rationale for the reduction in RPE65 expression. Increase in miR-410-3p expression was found to have a negative effect on EMT-associated genes, such as Snai1^34^. Concomitantly, inhibition of miR-410 was associated with increased expression of RPE65 and OTX2^35^. ROS feeding of ARPE-19 resulted in a significant increase in miR-410. There are some miRNAs among upregulated after ROS treatment that will promote autophagy and some that will suppress it via activation of mTOR. MiR-184 and miR-199a-3p, overexpressed after ROS treatment, promote RPE differentiation via suppression of AKT2/(mTOR) signaling pathway and activation of autophagy^36,37^.

When the dataset was analyzed with the clusterProfiler KEGG enrichment tool^38^ the most highly ranked KEGG pathway was that of osteoclast differentiation (data not shown). Though in much different locations, RPE and osteoclasts are both professional phagocytes and share certain signatures. It appears that feeding of ROS to ARPE-19 sets in motion a response paralleling osteoclast differentiation.

## Discussion

All current RPE cell models are known to be transcriptionally distinct from native RPE^10^. Despite this, there are many similarities: melanogenesis pathway genes in ARPE-19 cells grown in PYR are more similar to native tissue. Unfortunately, ARPE-19 and all other cultured RPE cells have much lower expression levels of many genes important in RPE metabolism and function, including visual cycle, lipid, vitamin and mitochondrial metabolism related genes, (see Supplementary Figure S12). In this regard, one of the most perplexing questions in RPE culture is the severe reduction in/absence of RPE65 transcription/translation in RPE cell models. Here, we addressed the main drivers of RPE65 regulation, comparing transcriptional profiles of native and cell culture models with various levels of RPE65 expression and performing experiments to study transcriptional and translational regulation of RPE65. We show that the metabolic and physiological status of ARPE-19 cells plays an important role in RPE65 expression, while its altered landscape for RPE65 transcriptional regulation also plays a major role.

It is difficult to determine based on our RNA-seq data which TFs may be involved in the upregulation of RPE65 expression in NAM-cultured ARPE-19, with ~ 9% of protein coding genes differentially expressed in NAM cultured cells. However, there is higher expression of two factors, FOSB and FOSL2, members of the AP-1 TF family triggered by diverse factors including cytokines, growth factors, oxidative stress, light damage, etc. For example, FOSB is known to be induced in ARPE-19 cells by oxidative stress^39^. Fos family TFs are also involved in mediation of light-induced damage in photoreceptors. Though the main AP-1 TF implicated is c-Fos, FosB can partially replace c-Fos^40^and AP-1 activity can be pharmacologically suppressed by corticosteroids to prevent light damage^41^. As the NAM media contains hydrocortisone, we see activation of corticosteroid-regulated genes along with activation of steroid biosynthesis pathway. However, possible interaction of hydrocortisone with the increased levels of Fos family genes is not clear in this regard and might mask slight changes in RPE65-modulating TFs.

While NAM can rescue RPE65 protein expression and promote cobblestone morphology of ARPE-19 cells^9^cells are less pigmented than those grown in PYR, consistent with reduced expression of the melanogenesis pathway genes TYR, TYRP1, and DCT (Supplementary Figure S4). In addition, Seahorse analysis showed that ARPE-19 cells grown on PYR exhibit higher glycolysis and mitochondrial respiration rate compared to NAM grown cells. Nicotinamide is a precursor of NAD + and NADP + cofactors essential for the tricarboxylic acid cycle and the mitochondrial electron transport chain. With respect to the higher mitochondrial respiration rate of PYR cells, Kang and Hwang reported that nicotinamide addition enhances mitochondria quality in human cells through activation of autophagy^23^. They showed that the effect of nicotinamide is mediated through an increase of the [NAD^+^]/[NADH] ratio and activation of SIRT1, finding that treating human fibroblasts with 5 mm NAM activates autophagy and causes a decrease in mitochondrial content and ROS levels, while increasing mitochondrial membrane potential^23^. NAM also induces transformation of mitochondria from filamentous network structures to short dot structures^23^perhaps enhancing overall mitochondrial quality by removing defective mitochondria. We hypothesize that NAM decreases mitochondrial respiration as well as ROS production in ARPE-19 cells leading to less stressed cells but with continuing high energy production. Thus, the high glucose level in PYR may not be as optimal as NAM for these cells. It is known that RPE cells in vivo use diverse energy sources, including lactate, succinate, and alpha ketoglutarate^42^and our results may be an in vitro reflection of that fact.

ARPE-19 cells are physiologically competent in a major in vivo role of RPE, that of daily trogocytosis or ingestion of photoreceptor outer segment membranes, often called phagocytosis^43^. We attempted to see if expression of RPE65 is modulated through feeding of ROS. However, RPE65 mRNA was reduced by about 40% in cells fed ROS, indicating a significant negative regulation due to phagocytosis. To further explore this, we interrogated effects of feeding ROS on expression of miRNAs in ARPE-19. Most of the important RPE-defining miRNAs, such as miR-204/211, miR-200 family, and miR-221/222^44^ were not significantly altered, indicating overall stability in the treated ARPE-19 cells. Also, when compared with the differential expression of miRs in differentiation of H9 ESCs to RPE^45^ only one miR (miR-214-3p) was shared in common, likely because the NAM cultured cells are already differentiated. With 6 miRNAs significantly downregulated and 34 significantly upregulated, out of over 1300 miRNAs detected, we conclude that ROS uptake has a rather precise effect on ARPE-19. Target analysis of the differentially expressed miRNAs showed effects on phagocytosis related genes and targeting of the Jak/STAT signaling pathway that is activated in response to photoreceptor damage; inhibition of JAK/STAT signaling can protect photoreceptors from degeneration^46^. MiR-184, another important RPE expressed miRNA, is significantly upregulated in ROS-treated cells. Upregulation of miR-184 in RPE is associated with upregulation of LAMP1, required for formation of phagolysosomes^47^. This may be a direct physiological effect of feeding ROS. Interestingly, miR-184 is downregulated in RPE cultured from AMD donors^47^. Inhibitors of miR-184 also negatively impacted ARPE-19 phagocytosis. This could be an indirect reason why RPE65 mRNA expression decreases with feeding of ROS. Significantly, we see upregulation in miR-206 and miR-410, implicated in regulation of SOX9 and OTX2 (seen to be decreased), TFs that regulate RPE65 and other visual cycle genes^11,33^providing a further rationale for reduced RPE65 expression. Increase in miR-410-3p expression was found to have a negative effect on EMT-associated genes, such as Snai1^34^. The PI3K/Akt anti-apoptotic pathway is enhanced by miR-126 expression, activating downstream Ras/Raf-1/ERK1/2 and PI3K/Akt signaling pathways by targeting and silencing SPRED1 and PIK3R2^48^. MiR-126 overexpression is associated with inhibition of oxidative stress and inflammatory response via activation of SIRT1/Nrf2 signaling in human umbilical vein endothelial cells^49^. MiR-126-5p promotes mitochondrial autophagy and is an upstream inhibitor of peroxisome proliferator-activated receptor-γ co-activator 1-alpha (PGC1α). The inhibition of PGC1α upregulates BCL2 and BCL2 interacting protein 3 (BNIP3), further activating a PRKN-independent selective mitochondrial autophagy pathway^50^. As discussed earlier, timely recycling of poorly functioning mitochondria is crucial for proper energy production in RPE. Although downregulation of miR-126 and miR-146-5p suggests pro-EMT changes, actual EMT is not implicated as seen by the lack of changes in miR-204/211, miR-200 family, and miR-221/222.

Enrichment analysis revealed that the KEGG pathway most affected was that of osteoclast differentiation. Both osteoclasts and RPE cells are professional phagocytes. Osteoclasts, required for bone remodeling, are of macrophage-derived lineage^51^. Osteoclasts share similarities with RPE, including expression of the same isoforms of MITF, most prominently MITF-D, that is different from other cell types such as melanocytes (predominantly MITF-M)^52,53^. Like in RPE, MITF is a very important TF in osteoclasts. MAP1LC3B (LC3)-related autophagy/phagocytosis is also important in both cell types. Thus, it appears that feeding ROS to ARPE-19 upregulates phagocytosis pathways, perhaps in common with “osteoclast differentiation”. It appears that induction of phagocytosis has a negative effect on the visual cycle and expression of RPE65. We are investigating this phenomenon further.

While we found that HEK 293T cells, a cell line of neuroepithelial origin^54^ widely used for heterologous protein production, can translate detectable amounts of RPE65 mRNA regardless of codon optimization, presence of 3’ UTR, or promoter used, while expression in other epithelial cells such as ARPE-19 or COS7 were greatly affected by codon optimization, use of promoter, or IRES elements. HEK293T cells still could notably increase protein production when codon optimized sequence was used. Surprisingly, we did not observe a significant difference in protein production in ARPE-19 or HEK293T cells between use of RPE65 ORF alone versus RPE65 + full length 3’ UTR. The difference in RPE65 expression in ARPE-19 between use or not of IRES (Supplementary Figure S6) was also surprising given questions about their functionality^25^. However, that difference may have been due to the different promoters used: human ferritin heavy chain (hFerH) promoter versus the CMV promoter.

When transduced with the same number of viral particles ARPE-19 cells grown on NAM contained 2 times more RPE65 mRNA than PYR cells. (Supplementary Fig. S8). It could be that NAM-grown cells are more easily infected compared to cells grown on PYR. Another explanation could be that RPE65 mRNA is degraded faster under the more oxidative conditions in PYR grown cells.

We conclude that the phenomenon of reduced RPE65 expression in culture is a multifactorial one involving primarily metabolic and transcriptional aspects, with translational factors playing a smaller role. It is not just RPE65 expression: many other RPE-signature genes also show reduced expression. There may be a cell-specific component here as cell lines such as HEK293 feature more robust expression of ORF constructs than the ARPE-19 cell line tends to achieve. Absence of in vivo molecular cues, such as from the photoreceptors, likely also play an important role in reduced RPE65 expression, though phagocytosis does not appear to be included among these.

## Methods

### Cell culture

Human ARPE-19 cells were grown on plastic coated with 1 mg/cm^2^ laminin (ThermoFisher cat #23017015) in pyruvate (PYR)^10,18^ and nicotinamide (NAM)^9^ media, following published protocols. For the PYR protocol, ARPE-19 cells were grown in Dulbecco’s modified Eagle’s medium (DMEM; ThermoFisher Scientific, Grand Island, NY) with 4.5 g/l glucose, L-glutamine, and 1 mM sodium pyruvate, supplemented with 1% fetal bovine serum (FBS), penicillin (100 U/ml), and streptomycin (100 µg/ml), as described previously^10^. For the NAM protocol, cells were first grown with the PYR protocol media for 1 week and then switched to MEM-alpha (ThermoFisher cat #42360032) supplemented with GlutaMAX, 1% FBS, 1% Penicillin/Streptomycin, 1% N1 supplement, taurine (0.25 mg/ml), hydrocortisone (20 ng/ml), triiodothyronine (0.013 ng/ml), and 10 mM nicotinamide. Laminin coating allows for faster differentiation and shortens culturing time from four months to one month. The cells were maintained at 37 °C in a humidified environment of 5% CO_2_. The cells were allowed to grow for 1 month with media exchange performed twice a week. HEK293T cells were grown in DMEM with 10% FBS and FreeStyle HEK293F cells (Invitrogen) were grown according to supplier’s protocol. COS7 cells were grown in DMEM GlutaMax with 10% FBS.

### Native human RPE tissues

All human specimens were obtained following a University of Maryland Baltimore Medical Center institutional review board (IRB) approved exemption for anonymized tissue samples for scientific use. Informed consent was not required. Eye tissues used for this purpose were stripped of identifiable information except for sex and age. Postsurgical tissues were taken directly from the operating room on ice. Times from enucleation to RNA extraction were ≤ 24 h.

We confirm that all experiments were performed in accordance with relevant guidelines and regulations. IRB review determined that it met the definition of Not Human Subjects Research (NHSR), that oversight was not required, and the need to obtain informed consent was waived by the University of Maryland Baltimore Human Research Protections Office Institutional Review Board.

### Western blotting

Denatured lysates in 4 × LDS buffer were separated on 4–12% SDS-PAGE gels (Invitrogen, ThermoFisher) and electro-transferred (iBlot2; ThermoFisher Scientific). Membranes were blocked with Odyssey blocking buffer (LI-COR Biosciences). Secondary antibodies IRDye 680RD and IRDye 800CW were used for visualization on an Odyssey Infrared Imager (LI-COR Biosciences). The primary antibodies used were as follows: α-Tubulin mouse monoclonal antibody (Li-COR), β-Actin rabbit monoclonal antibody (Li-COR), β-Actin Mouse Monoclonal (Li-COR), mouse anti-RPE65 (1:200) custom-made; rabbit anti-RPE65 antibody (1:8,000) custom-made; rabbit anti-LRAT custom-made antibody (1:2,000); mouse anti-GAPDH GenTex (1:10,000); mouse anti-BEST1 (1:5,000) Abcam: ab2182, Rabbit monoclonal RPE65-[EPR7024(N)]-C-terminal antibody (1:2000) (Abcam, Cat. No. ab175936). Original blots/gel are shown in Supplementary Figure S13 (for Fig. 3) and Supplementary Figure S14 (for Supplementary Figures S3, S6, and S8).

### Immunoprecipitation and proteomics analysis

NAM-differentiated ARPE-19 cells were probed for RPE65 by immunoprecipitation (IP) using commercially available rabbit monoclonal RPE65 antibody (Abcam, Cat no. ab175936). RPE microsomes extracted from bovine eyes and enriched in RPE65 protein were used as a positive control. RPE65 antibody was covalently immobilized to Dynabeads^®^ M-270 beads (3.3 µg Ab per mg of Dynabeads) using Dynabeads^®^ antibody coupling kit (Cat. No. 14311D, ThermoFisher Scientific, Waltham MA). For immunoprecipitation, all the steps were carried at 4 °C. The cells were washed with ice-cold 1X PBS buffer and lysate was prepared in ice-cold lysis buffer (buffer A) containing 25 mM Tris buffer pH 7.4, 150 mM KCl, 5 mM EDTA, 0.5% NP-40 and protease inhibitor cocktail (Roche Diagnostics). The pre-cleared cell lysate containing ~ 1 mg of total protein was added to 100 µl of the antibody-coupled beads in the microfuge tube and incubated for 18–24 h with orbital rotation at 4 °C. The tubes were then placed on a magnet for 1 min and the supernatant was carefully removed. The tube was removed from the magnet and the beads containing Ab-protein complex were washed three times with buffer A, 5 min incubation each, and the bound protein was eluted by incubating with 50 µl of elution buffer for 10 min at room temperature. The eluate was collected and transferred to a fresh tube. An aliquot of 5 µl of IP eluate was subjected to immunoblotting for RPE65 protein and the remaining volume was used for mass spectrometry. Immunoprecipitation samples were sent for proteomics analysis (Poochon Scientific, Frederick, MD). Samples were processed for trypsin/LysC digestion, concentrated and desalted, and analyzed on a 110 min gradient LC/MS/MS run on a Thermo Scientific Exploris 240 mass spectrometer. Raw data files were compared against human or bovine databases, as required, using Proteome Discoverer 2.4 software (Thermo Scientific).

### RNA sequencing and data processing

Total RNA extraction from 3 replicates of ARPE-19 cell cultures differentiated under NAM or PYR protocols was done using Maxwell RCS simplyRNA Cells Kit (cat #AS1390). The RNA samples were submitted to Novogene (Durham, NC) for sequencing (2 × 150 bp paired end protocol on Illumina HiSeq 2500). Short reads were mapped on the GRCh38.p5 human genome. DESeq2 was used to identify differentially expressed transcripts^55^. Enrichr was used for pathway analysis (https://maayanlab.cloud/Enrichr/)^56^.

The same RNA samples were used for preparation of Nanopore libraries using LSK-PCB109 kit (Oxford Nanopore Technologies (ONT), Oxford, UK) according to manufacturer’s protocol and sequenced on one MinIon flowcell (FLO-MIN106D; ONT). Nanopore reads were base-called with Nanopore Guppy in high accuracy mode and transcripts mapped with minimap2 to human genome^57^. For isoform discovery, we first used Pychopper v2 (https://github.com/epi2me-labs/pychopper) to identify and orient full length reads cDNA reads. Those reads were subjected to the pinfish pipeline (https://github.com/nanoporetech/pipeline-pinfish-analysis). Briefly, reads were mapped with minimap2, consensus clusters were found, polished, and partials were collapsed to remove RNA degradation products. The final polished and collapsed transcripts were visualized in IGV. Subread was used for counting fragments^58^.

RNA extraction with nucleus and cytoplasm fractionation was done using the Ambion™ PARIS kit.

### Constructs and plasmids

The human RPE65 ORF + 3’ UTR clone (NM_000329.2) was purchased from Creative Biogene. Mutations in the RPE65 ORF were corrected using a Quickchange kit (Agilent, San Diego). TYR and NEAT genes were amplified from ARPE-19 cDNA. Cloning was done using Gibson assembly. The constructs RPE65 + 3’ UTR full cDNA, RPE65-3’ UTR, RPE65 ORF, and RPE65 + partial 3’ UTR were cloned into the pcDNA3 vector and pVitro2 vector. All primers used for cloning are in listed in Supplementary Table S4.

### Transfections of ARPE-19 cells

ARPE-19 cells are difficult to transfect compared to HEK293T or HEK293F cells and special procedures had to be developed. For successful transfection, ARPE-19 cells cannot be in culture more than 3 weeks (no tight junctions should be formed during culturing to preserve transfectability). The best results were obtained with cells freshly cultured from liquid nitrogen cryostorage. ARPE-19 cells were seeded the day before transfection at a density of 1.5 × 10^6^ cells in 9.6 cm^2^ dishes to reach 40–60% confluency on the day of transfection. FuGENE^®^ 6 (Promega Corporation) was used as transfection reagent at 3:1 ratio.

### Pseudovirion production

Pseudovirions were produced by co-transfection of cells with packaging plasmid psPAX2, pLenti-GFP transfer plasmid containing RPE65 and pLenti, and plasmid encoding VSV-G fusion-protein. We used Expi293F™ cells (cat #A14527, ThermoFisher) following the manufacturer’s protocol, using the same ratio of plasmids (3:2:2) with a total of 30 mg DNA/30 ml cell culture using 293fectin™ Transfection Reagent (ThermoFisher). For HEK293T adherent cells we used the same ratio of plasmids (3:2:2) with a total of 30 µg per 2 × 10^6^ cells for transfection using FuGENE^®^ 6 Transfection Reagent (Promega Corporation). The media supernatants were harvested at 48 h post transfection and centrifuged at 800 × g for 5 min, and then passed through a 0.45 μm filter. Pseudotyped virus stocks were aliquoted and stored in cryovials at −80 °C.

### Measurement of physical and infectious viral titer

ARPE-19 cells were seeded at 1.5 × 10^6^ in 12 well plates and infected with serial dilutions of pseudotyped virus. At 48 and 72 h after transduction the percentages of GFP positive cells were measured and cells were collected for RNA and protein level quantification. To measure physical titer, we used p24 and qPCR assays as described^59^.

### Seahorse assay

For Seahorse assays, ARPE-19 cells were differentiated on Agilent Seahorse XF24 cell culture plates (cat#100777-004) without laminin according to the NAM or PYR protocols. Mito Stress Test kit (103015-100) was used according to the manufacturer’s protocol. The protocol involves 3 serial injections of oligomycin (inhibitor of ATP synthase (complex V) to assay cellular ATP production), FCCP (uncoupling agent to calculate spare respiratory capacity), and rotenone/antimycin A (complex I and III inhibitors to calculate non-mitochondrial respiration). We determined the optimal final concentration of oligomycin to be 2.5 mM, FCCP 1 mM and Rot/AA 1 mM. The base assay media contained 10 mM glucose, 1 mM pyruvate, and 2 mM glutamine; the PYR protocol assay media contained 25 mM glucose, 2 mM pyruvate, and 2 mM glutamine; and the NAM protocol assay media contained 10 mM glucose, 1 mM pyruvate, 2 mM glutamine, and 10 mM NAM.

### Ribosomal profiling and RNA fractionation

Differentiated ARPE-19 cells were used for polysome profiles as described^60^. Briefly, ribosomes were stalled by adding cycloheximide (CHX; 100 µg/ml) to the cells and after 10 min of exposure cells were washed on ice 3 times with PBS with 40 U/ml Ribonuclease Inhibitor + 100 µg/ml CHX. Cells were lysed by Nitrogen Decompression using a prechilled 4639 Cell Disruption Vessel (Parr) for 10 min at 700 psi in buffer 1 (50 mM HEPES, pH 7.0, 150 mM NaCl, 1.5 mM MgCl_2_, 0.5% NP-40, 2X EDTA-free protease inhibitors, 4.0 U/ml Ribonuclease Inhibitor). 10 OD_260_ units of cell lysate, cleared by centrifugation, was layered on 7–47% sucrose gradients, made using a Gradient Master (Biocomp), and ultracentrifuged at 33,000 rpm for 2 h and 50 min at 4 °C in a Beckman SW41 rotor. One ml fractions were collected from the completed gradients using a programmable density gradient fractionation system spectrophotometer (Foxy Jr. model; Teledyne Isco, Lincoln, NE). RNA was purified from 500 µl of each fraction using the Maxwell 16 LEV simplyRNA kit on the Maxwell 16 instrument (Promega), with DNase I treatment. RNAs were quantitated by quantitative reverse-transcription (qrt-)PCR and using transcript-specific primers (Supplementary Table S4). For calculation of percentage of mRNA in each fraction ΔΔCt values were used as described in data analysis, data visualization and statistical analysis section.

### Rod outer segments (ROS) extraction and feeding

Rod outer segments (ROS) were prepared from bovine retinas. 200 retinas were vigorously shaken in 180 mL sucrose-containing buffer (45% sucrose in buffer A: 100 mM potassium phosphate, pH = 7.0, containing 1 mM MgCl_2_, 0.5 mM DTT, and 0.1 mM EDTA). This was followed by centrifugation to obtain a crude ROS fraction (3,000 x g, 5 min, 4 °C). The supernatant was filtered through gauze and diluted 1:1 with buffer A. The preparation was again centrifuged for 7 min at 4,400 x g. Each pellet was resuspended in 1 mL of sucrose buffer A (ρ = 1.105, 26.3%). The ROS were purified by discontinuous density gradient centrifugation (9 mL of sucrose-buffer (ρ = 1.135, 34.2%), then 8.5 mL of sucrose buffer (ρ = 1.115, 28.7%) and crude ROS equally distributed among the tubes by layering 3 mL on the top of each. The tubes were centrifuged in a swinging bucket rotor (SW 32 Ti, Beckman) for 1 h at 27,000 x g and 4 °C without brake. The ROS is collected as a slightly orange band at the 1.115–1.135 interface. The ROS are diluted 1:1 with buffer A and harvested by centrifugation in a fixed angle rotor (TLA 120.2, Beckman) at 39,000 x g for 1 h at 4 °C and the pellet stored at −80 °C. Before use, each tube of ROS was resuspended in 250 µl of DMEM media with high glucose, 6 tubes were combined and counted on Nexcelom cell counter.

Aliquots of ROS in DMEM, high glucose (1 × 10^7^ ROS/mL, 625 µL per 10 mL of NAM-MEM media) were added to the ARPE-19 culture plates grown on Corning BioCoat Laminin plates (Corning) for 2 months in NAM-MEM media. After 5 h, fresh NAM-MEM media (10 mL) was added, and the cells collected after 24 h. Cells were extensively washed with fresh NAM-MEM media (3x), and PBS (3x) before adding 720 µL of homogenization buffer with 2% thioglycerol (AS1460, Promega) to each plate. Cells were scraped with a cell scraper, collected, and stored at −80 °C. Total RNA was isolated from 200 µL of suspended cells by Maxwell^®^ RSC miRNA from Tissue kit according to manufacturer’s recommendations. Small RNA library preparation (NovaSeq SE50) and analysis were done by Novogene.

### Data analysis, data visualization and statistical analysis

GraphPad Prism was used for data visualization and statistical analysis. Unpaired t test was used to determine differences in RPE65 expression. Standard curve and relative expression qPCR data was analyzed on QuantStudio.

The calculations of the percent RNA in each sucrose gradient fraction are as follows:

$$\Delta {\text{CX}} = {\text{CTX}} - {\text{CT1}}$$

$$\% ~{\text{RNA}} = {\text{2}} - \Delta {\text{CTX2}} - \Delta {\text{CT1}} + {\text{2}} - \Delta {\text{CT2}} +\cdots + {\text{2}} - \Delta {\text{CTY}} * {\text{1}}00$$

X = the number of the fraction that is calculated; Y = the total number of fractions.

Li-COR ImageQuant software was used for western blot data visualization and quantitation and normalized to chosen internal loading control.

Transcripts were visualized in IGV. Human GRCh38 genome was used as a reference, gff3 of the final polished and collapsed transcripts from Nanopore data were loaded as separate tracks.

Targets of miRs were analyzed using miRTargetLink 2.0^26^. The clusterProfiler^38^ software was used for gene enrichment analysis, including GO Enrichment and KEGG Enrichment.

## Supplementary Information

Below is the link to the electronic supplementary material.

Supplementary Material 1

Supplementary Material 2

## Data Availability

Sequencing data are available at https://www.ncbi.nlm.nih.gov/bioproject/PRJNA1173186.
