# Supplementary material for: Regulation of RPE65 expression in human retinal pigment epithelium cells
Source: Sci Rep. 2025 Jul 25;15:27106. doi: 10.1038/s41598-025-12926-3 (PMC12297382; doi:10.1038/s41598-025-12926-3)

Supplementary files for:

REGULATION OF RPE65 EXPRESSION IN HUMAN RETINAL PIGMENT EPITHELIUM CELLS

Olga A Postnikova[1,4], Samuel William[1] , Sheetal Uppal[1], Steven L. Bernstein[2], Eugenia Poliakov[1], Igor B. Rogozin[3,5], and T. Michael Redmond[1]*

[1]Laboratory of Retinal Cell & Molecular Biology, National Eye Institute, NIH, Bethesda, MD 20892, USA.
[2]Departments of Ophthalmology and Visual Sciences, and Anatomy and Neurobiology, School of Medicine, University of Maryland, Baltimore, MD 21201, USA.
[3]National Center for Biotechnology Information, National Library of Medicine, National Institutes of Health, Bethesda, MD 20892, USA.
[4]Present Address: USDA-ARS, NEA, BARC, Animal Biosciences and Biotechnology Laboratory, Beltsville, MD 20705, USA.
[5]Present address: Life Science Research Centre, Faculty of Science, University of Ostrava, 710 00 Ostrava, Czech Republic.

Supplementary Table S1: Mass spectrometric detection of RPE65 peptides

	Accession	Gene	Protein Name	Origin	Description	Coverage [%	# Peptides	# PSMs	Abundance: F6: Sampl	# Unique Peptid	# AAs	MW [	calc. pI
Bovine Microsome	Q28175	RPE65	Retinoid isomerohydrolase	Bos taurus	Retinoid isomer	71	33	160	176301673.5	33	533	60.9	6.44
ARPE-19 IP	Q16518	RPE65	Retinoid isomerohydrolase	Homo sapiens	Retinoid isomer	31	11	19	5861356.734	11	533	60.9	6.48

Supplementary Table S3: Confirmation of RNAseq results by qPCR

	ERF	SFRP5	PER1	BMPR1B	CDH16	AKNA	TFCP2L1	R
qPCR	-1.10587	-2.03563	-1.92004	3.29275	-5.77454	2.497917	-6	
Nanopore	-4.81476	-3.772	-6.12519	NA	NA	NA	NA	
Illumina	-2.59304	-2.90031	-4.1212	2.73619	-6.43034	1.363254	-6.45443	0.986485

Supplementary Table S4: Primers used

A. qPCR primers for RPE65 gene

RPE65 gene region	Probe	Primer 1	Primer 2
RPE65 exon 7	5'/56FAM/ccactgcaa/zen/gcagtttt ggt/31ABkFQ/3'	cccaaagactccatgaagaaag	gcctacaacattgtaaagatccc
RPE65 exons 10-11	5'/56FAM/acgctcccca/zen/ataca actgcca/31ABkFQ/3'	gctccccaacctgaagttag	gatagtctcgtcactgcacag
RPE65 exons 13-14	hs01071462 (Custom predesigned primer set)		

B. Cloning primers

Cloning Primer	Oligo original sequence (uppercase = gene-specific primer)
utr-64-F	aacgggccGATTGCAGACCTGAAGCTGATTTTCTC
utr-64-R	ctgcaatcGGCCCGTTTAAACCCGCTG
pcdna-utr-F	ccaagctggctagcaATGAGCATCCAGGTGGAAC
pcdna-utr-R	tcttgctggagtatgcTCACTAGGACTTCTTGAACAG
rpe65-F	CTCGAGCACCACCACCAC
rpe65-R	GGGTGGTGGCCATATTATC
rpe65-utr-F	CTCGAGCACCACCACCAC
rpe65-utr-R	GGGTGGTGGCCATATTATC
pvitro2-F	aacagtcagtTCCTAGGGTCGACAATCG
pvitro2-R	gaccctaggaACTGACTGTTTGACAATTAATCATC

C. qPCR primers

Gene	Probe	Primer 1	Primer 2
HPRT1	/56-FAM/AG CCT AAG A/ZEN/T GAG AGT TCA AGT TGA GTT TGG /3IABkFQ/	GCG ATG TCA ATA GGA CTC CAG	TTG TTG TAG GAT ATG CCC TTG A
MERTK	/56-FAM/TG CAG CAT T/ZEN/C AGG TCA AGG AAG CT/3IABkFQ/	GAT GTG GTA AGG CAG AGG TG	ACA CAG CAT TCT GAT CTC CTG
MITF	/56-FAM/TG AAA ACC G/ZEN/A CAG AAG AAA CTG GAG CA/3IABkFQ/	CTC GAG CCT GCA TTT CAA G	GTG GAC TAT ATC CGA AAG TTG C
OTX2	/56-FAM/AA ACC ATA C/ZEN/C TGC ACC CTC GAC TC/3IABkFQ/	CAT TCT GCT GTT GTT GCT GTT	CCA GAC ATC TTC ATG CGA GAG
FOSL2	56-FAM/AA TTT CTG C/ZEN/T GGC CGC CGC /3IABkFQ/	GGA TGA ATG CAC TGC CTG A	GGG AAC TTT GAC ACC TCG T
SOX9	56-FAM/AA GGG CCG C/ZEN/T TCT CGC TCT /3IABkFQ/	CGT TCT TCA CCG ACT TCC TC	CTG GGC AAG CTC TGG AG

Supplementary Figure S1. Correlation plot. Scatter plot graphs of FPKM values with Pearson correlation coefficients and p-values. RNA-seq data was obtained from: ARPE19 4 days, undifferentiated ARPE-19; ARPE19 4 Month, ARPE-19 cells differentiated for 4 months under PYR protocol; H1, RPE differentiated from Human H1 Embryonic Stem Cell (ESC) line; H9, RPE differentiated from Human H9 Human ESC line; HF, Human Fetal RPE; RPE\CHOR, RPE from nasal (NAS), temporal (TMP), and macular (MAC) regions of human RPE/choroid; RPE\Chor Bos, native Bovine RPE + choroid; RPE\Chor 4 weeks, 4 weeks old primary bovine RPE + choroid cells; and RPE \Chor, 8 weeks, 8 weeks old primary bovine RPE + choroid cells.

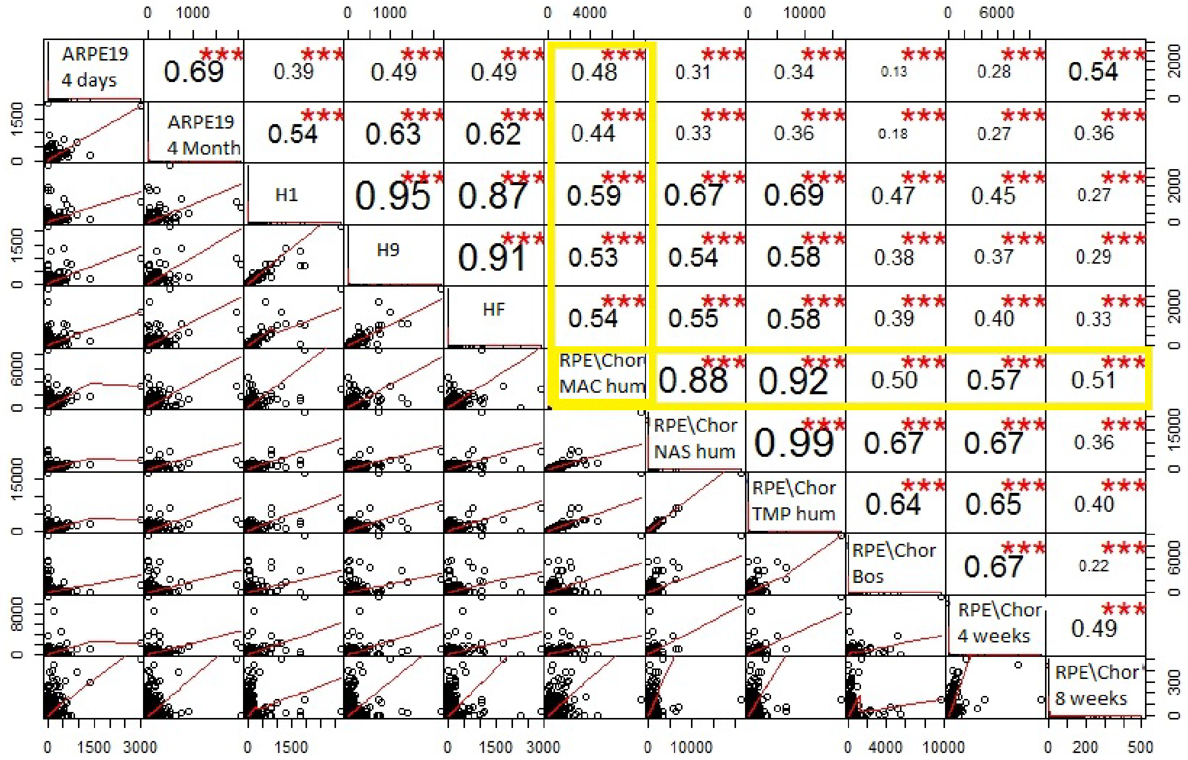

Supplementary Figure S2: Pearson (P) and Spearman (S) correlation coefficients of comparisons between different RPE models. ARPE19_4M**,** ARPE**-**19 cells differentiated for 4 month under PYR protocol; H1, RPE differentiated from Human H1 Embryonic Stem Cell (ESC) line; H9, RPE differentiated from Human H9 Human ESC line; HF, Human Fetal RPE; RPE\CHOR, RPE from nasal (NAS), temporal (TMP), and macular (MAC) regions of human RPE/choroid.

P \ S	ARPE_4M	H1	H9	HF	RPECHOR _MAC	RPECHOR _NAS	RPECHOR _TMP
ARPE_4M	1.00	0.88	0.89	0.90	0.79	0.80	0.80
H1	0.54	1.00	0.98	0.96	0.81	0.83	0.83
H9	0.62	0.95	1.00	0.96	0.81	0.82	0.82
HF	0.60	0.90	0.91	1.00	0.81	0.82	0.82
RPECHOR_MAC	0.46	0.49	0.47	0.45	1.00	0.99	0.99
RPECHOR_NAS	0.36	0.58	0.49	0.49	0.88	1.00	0.99
RPECHOR_TMP	0.39	0.60	0.52	0.51	0.93	0.99	1.00

Supplementary Figure S3: A, Western blots of ARPE-19 cells differentiated under PYR protocol (1) and RPE differentiated from iPSC (2); bovine microsomes (3) were used as a positive control for RPE65 and LRAT proteins. Mouse custom monoclonal anti-RPE65 (red) was used as primary; β-actin (green), loading control. ARPE-19 or RPE differentiated from iPSCs are positive for BEST1 and LRAT proteins, but no signal was observed for RPE65 using mouse custom monoclonal anti-RPE65 antibody. The original blots/gels are presented in Supplementary Figure S14; **B**, qPCR of genes found to be differentially expressed between ARPE-19 cells grown in either the NAM or PYR protocols.

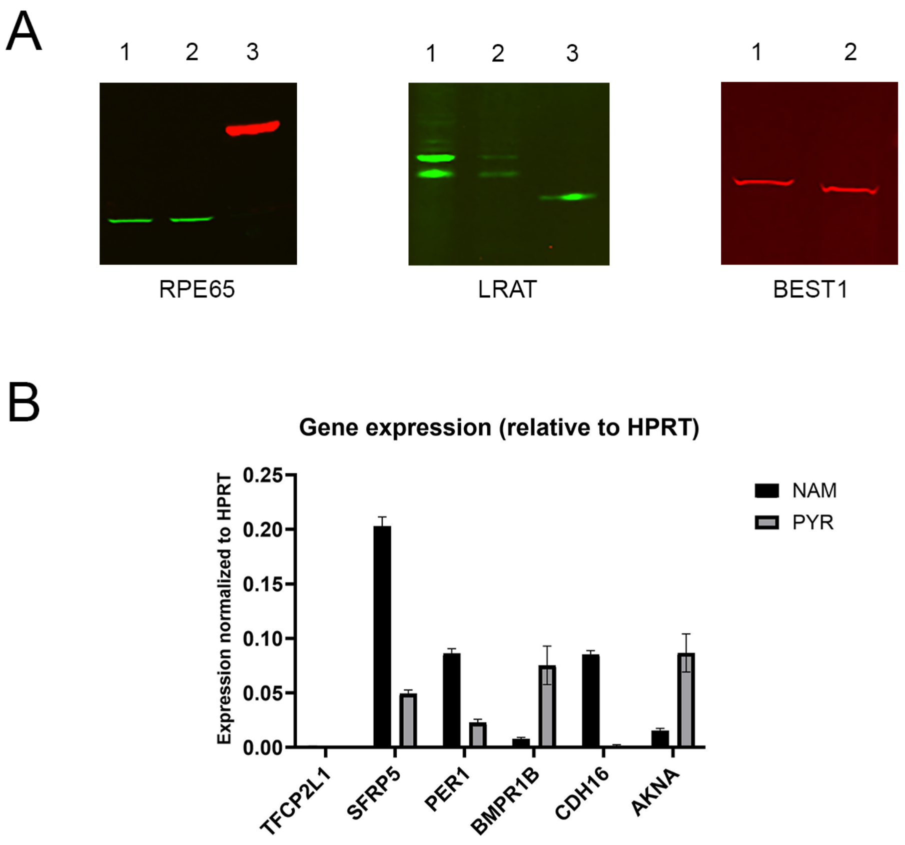

Supplementary Figure S4: Melanogenesis pathway. Green depicts genes downregulated on NAM protocol compared to PYR protocol.

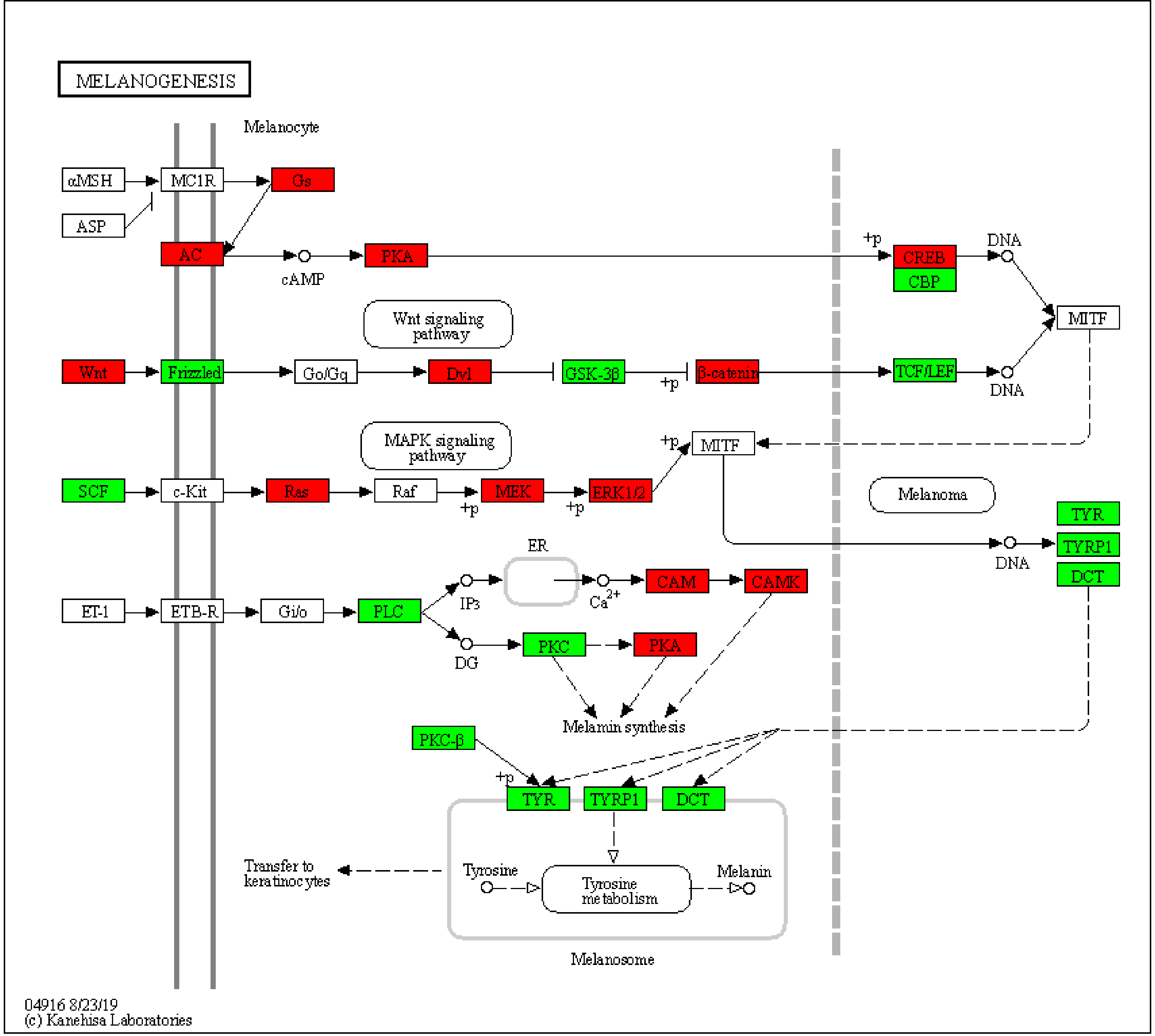

Supplementary Figure S5: Sterol Biogenesis pathways. Red represents up-regulated genes in ARPE-19 on NAM protocol compared to cells grown on PYR protocol

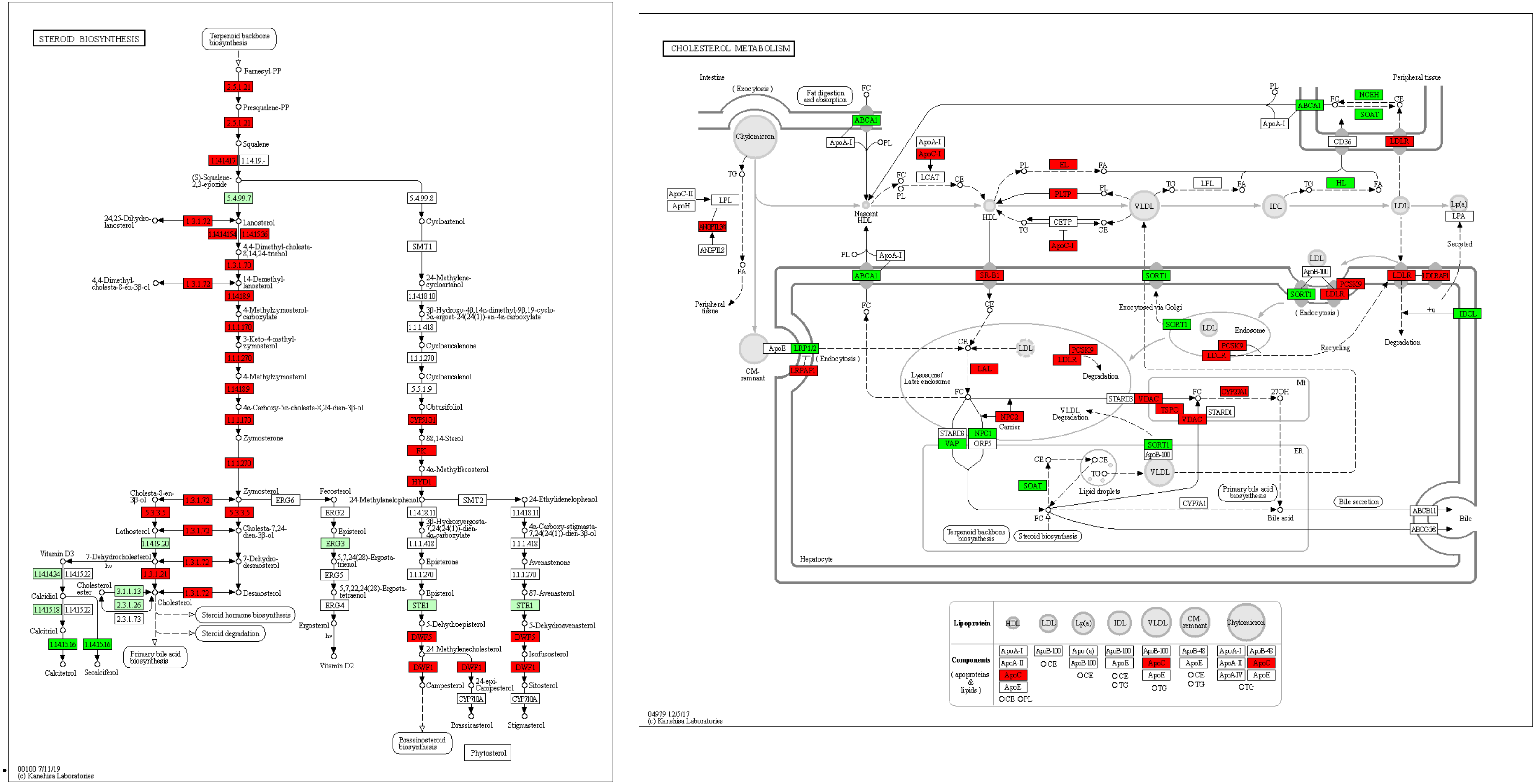

Supplementary Figure S6: A. RPE65 immunoreactivity probed in COS7 and HEK293T cells transfected with various RPE65 constructs. B. RPE65 immunoreactivity probed in ARPE-19 cells transfected using pcDNA (->CMV-promoter->t7-promoter->RPE65 mRNA->polyA signal) and pVitro2 (pV2; ->hFerH promoter->mEF1 5'UTR-> RPE65 mRNA ->FMDV IRES) vectors. B. Quantification of RPE65 protein expression in constructs with and without an IRES(LICOR). Blots were probed for RPE65 immunoreactivity using rabbit monoclonal antibody. The original blots/gels are presented in Supplementary Figure S14.

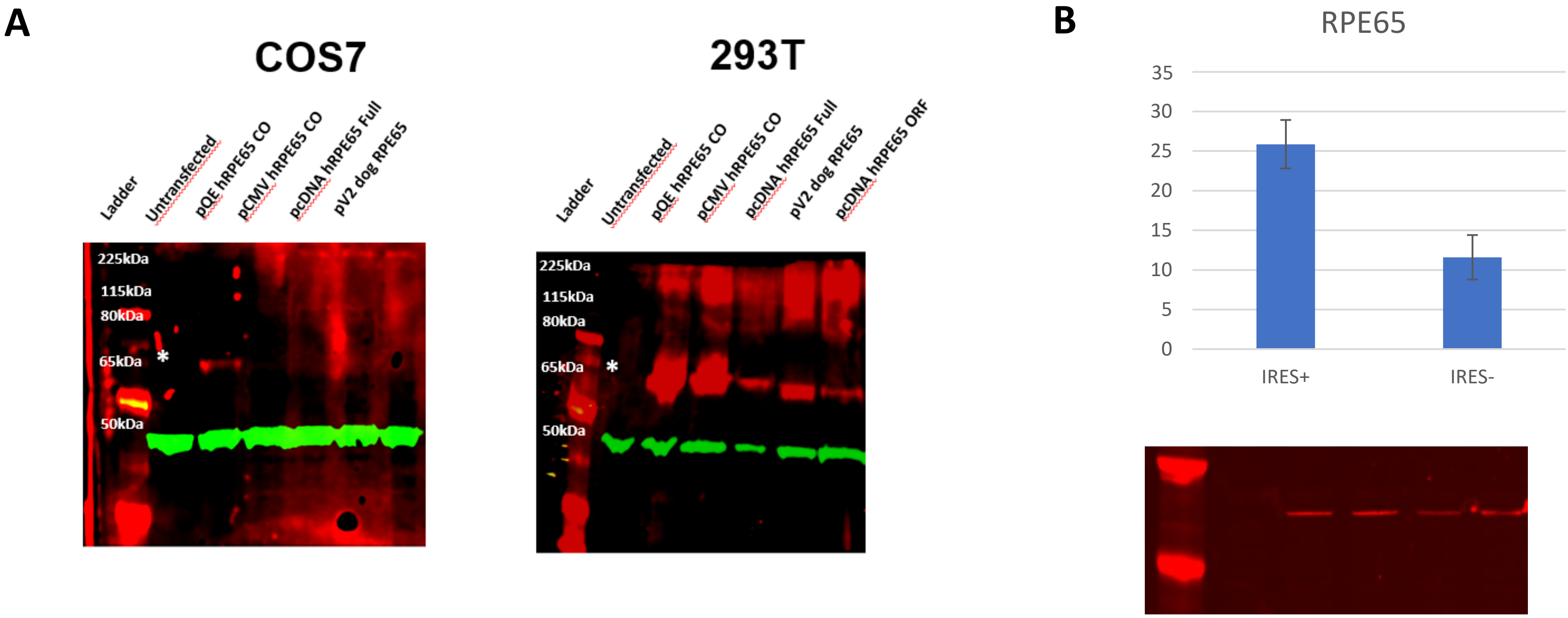

Supplementary Figure S7: Transduction of ARPE-19 with viral particles pseudotyped with VSVG spike protein and carrying pLenti vector with GFP.

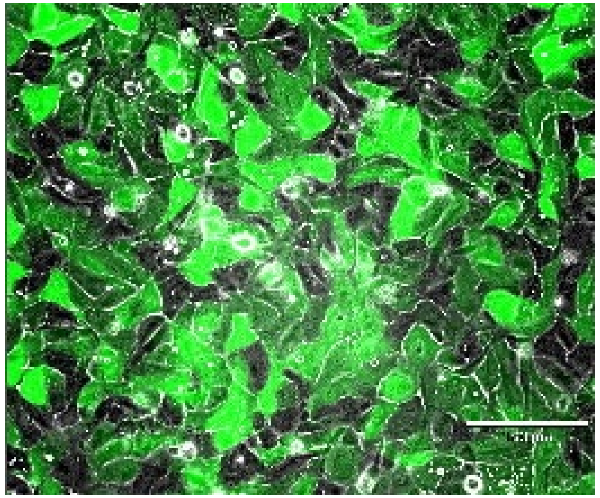

Supplementary Figure S8: Quantification of RPE65 mRNA in ARPE-19 cells grown on NAM or PYR media transduced with equal amount of pseudotyped viral particles carrying pLenti - full length RPE65. a. qPCR b. Detection of RPE65 protein with RPE65-[EPR7024(N)]-C-terminal antibody (Abcam, Cat. No. ab175936) in ARPE-19 cells transduced with pLenti-RPE65 full length mRNA. Arrow points at positive control (PC) band level at lower image display settings. Negative control (NC) was untransduced ARPE-19 cells. RPE65 is in green, Normalization control beta-actin is in red. The original blot/gel is presented in Supplementary Figure S14.

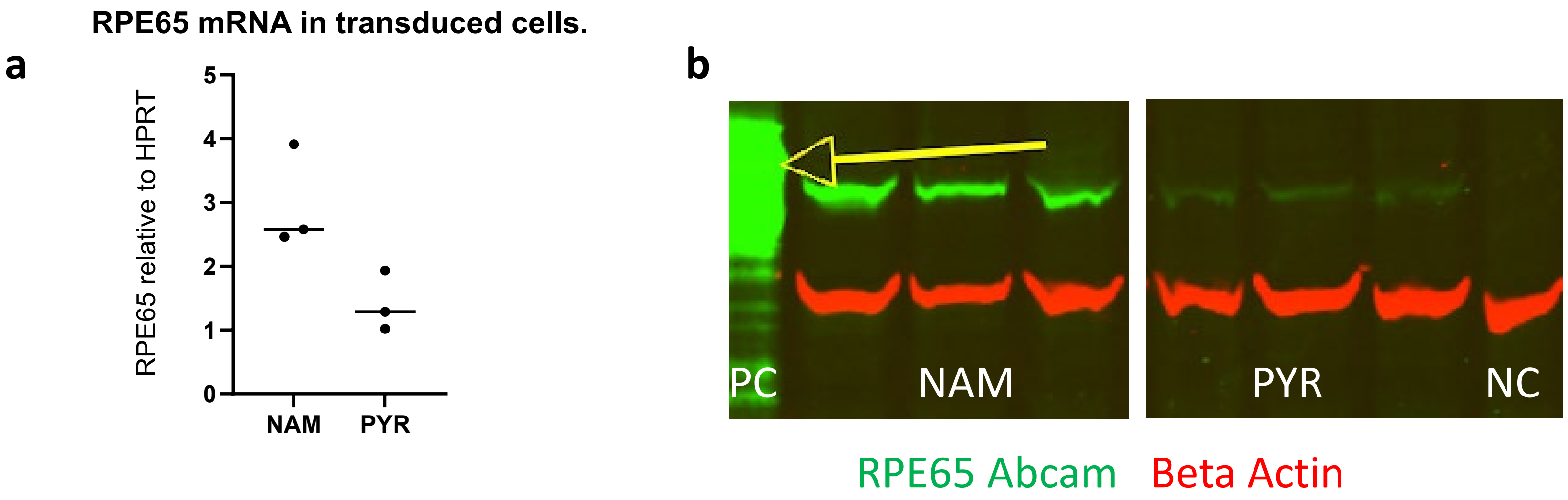

Supplementary Figure S9: The ratios between polysome fractions and all other fractions (free plus 60S and 80S). RPE65 mRNA levels were measured by qPCR with TaqMan primers in exons 13-14 or exons 10-11. Distributions (percentage) are of RPE65 and TYR mRNAs in each fraction during ribosomal profiling on 7-47% sucrose gradients. Protein-coding mRNA TYR is used as a positive control. RPE65 mRNA levels were measured by qPCR with TaqMan primers in exons 6-8 (skipped exon), exon 13-14 or exons 10-11. Fractions 1-6, free mRNP, fraction 7-9, 60S ribosomal subunit, fraction 9, monosomes, and fraction 10-14, polysomes.

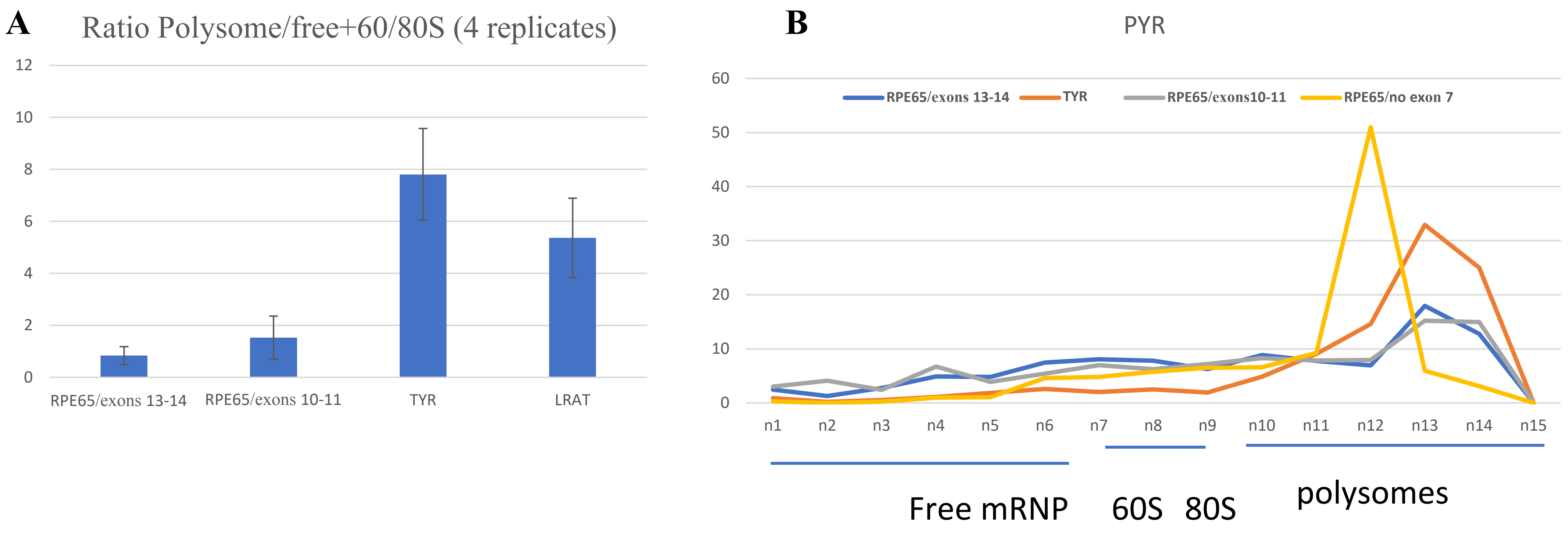

Supplementary Figure S10: Distribution of RPE65 mRNA in cytoplasm and nucleus. ARPE-19 cells were fractionated between cytoplasmic and nucleus fractions and RNA was extracted. qPCR was performed with GAPDH as a control for cytoplasmic localization and U2 for nucleus localization. All extractions were performed in triplicates. Percentage of each mRNA in nucleus (orange) and cytoplasm (blue) are presented in the graphs.

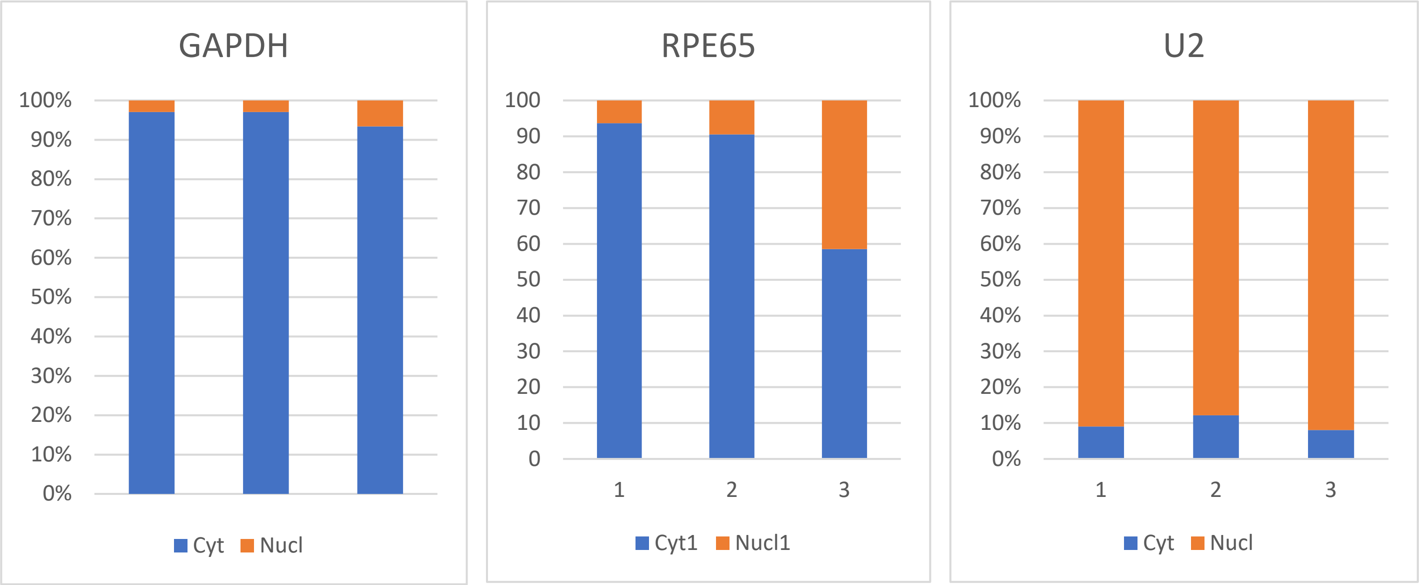

Supplementary Figure S11: Feeding of ROS decreases expression of transcription factors that may affect expression of RPE65. NAM differentiated ARPE19-cells were fed ROS and collected 24h after feeding. RNAs were isolated and subjected to quantitative rtPCR using primers for the indicated genes; *=p<0.1, **=p<0.01, n=3.

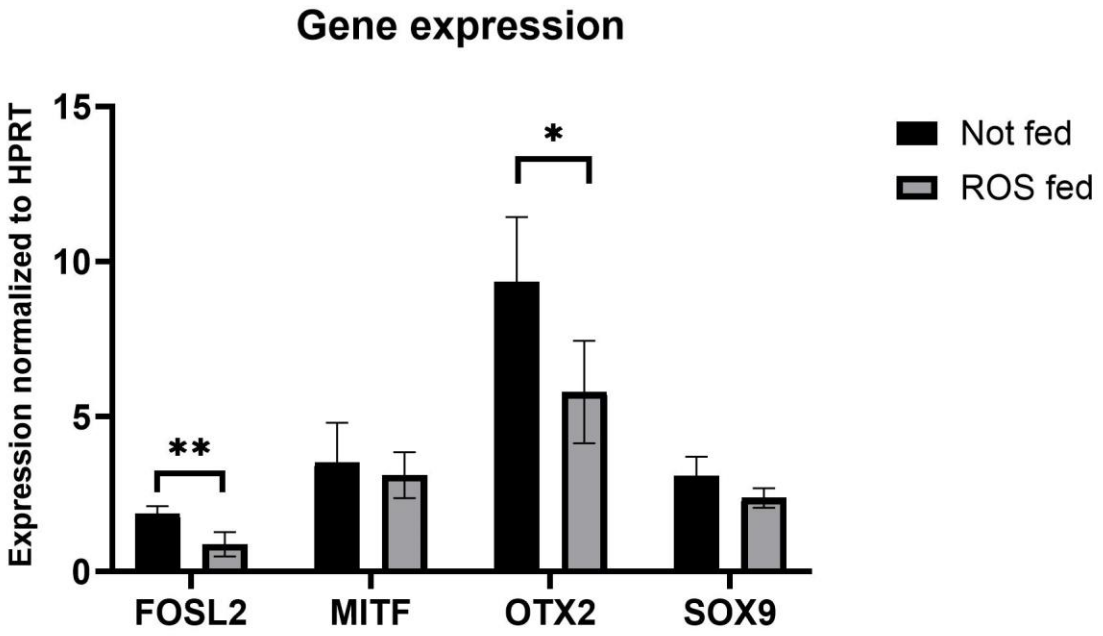

Supplementary Figure S12: Comparison of over-represented GO processes between ARPE-19, human fetal (HF) and native human macula RPE + choroid (RPE\CHOR macular).

Overrepresented GO biological process in 1000 most higly expressed genes	ARPE19	HF	RPE\CHOR Macular
translational elongation	6.80E-59	2.00E-61	3.90E-64
translation	3.30E-41	1.30E-33	2.80E-36
generation of precursor metabolites and energy	2.10E-14	2.50E-08	2.00E-21
oxidative phosphorylation	6.40E-09	3.20E-06	2.20E-15
electron transport chain	1.00E+00	1.00E+00	7.90E-10
cellular respiration	1.00E+00	1.00E+00	2.30E-07
mitochondrial electron transport, NADH to ubiquinone	1.00E+00	1.00E+00	4.80E-07
mitochondrial ATP synthesis coupled electron transport	1.00E+00	1.00E+00	1.00E-06
response to inorganic substance	1.00E+00	1.80E-06	9.40E-07
anti-apoptosis	1.00E+00	1.20E-07	7.00E-06
response to organic substance	1.00E+00	7.80E-06	4.00E-09
negative regulation of protein metabolic process	1.00E+00	1.00E+00	6.70E-09
oxidation reduction	3.30E-07	1.00E+00	8.00E-06
intracellular transport	2.30E-09	9.90E-09	1.00E+00
intracellular protein transport	2.60E-08	1.60E-07	1.00E+00
cellular protein localization	4.00E-08	1.90E-07	1.00E+00
cellular macromolecule localization	4.80E-08	1.60E-07	1.00E+00
protein localization	1.50E-06	4.70E-06	1.00E+00

Supplementary Figure S13: Original blots/gels for Figure 5.

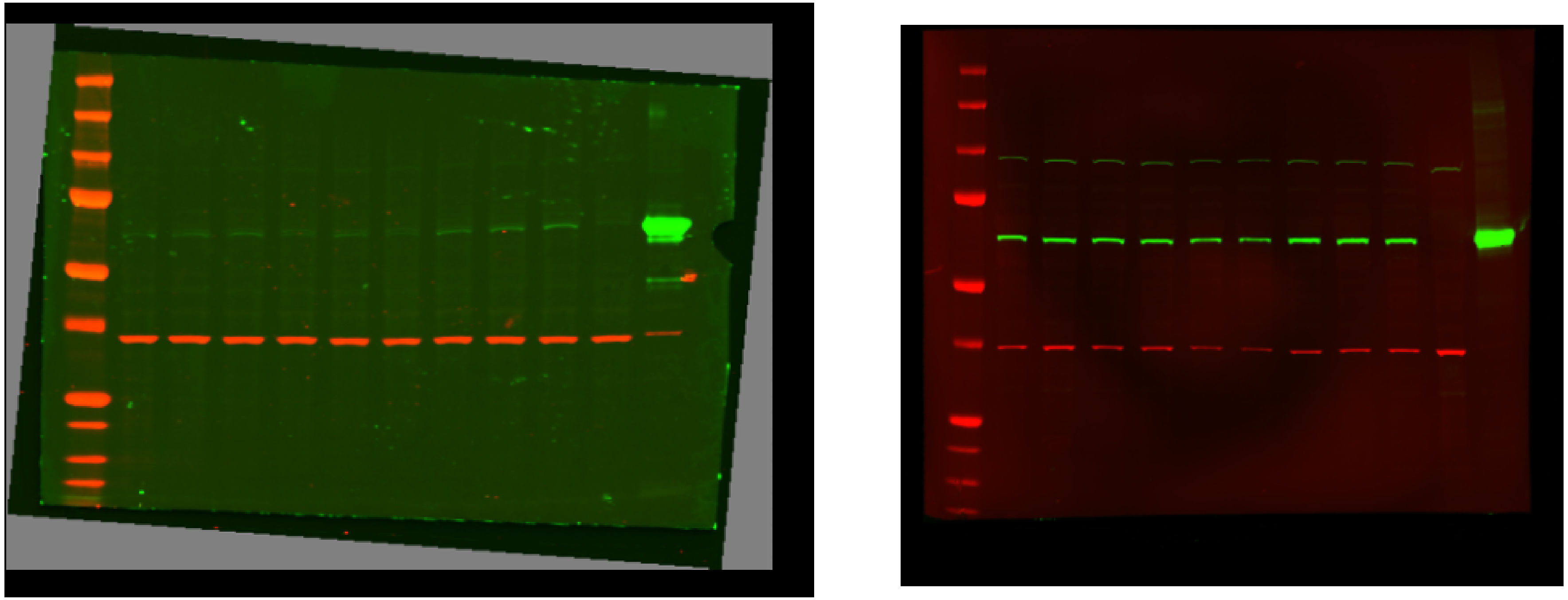

Supplementary Figure S14: Original blots/gels for supplementary figures. A, Supplementary Figure S3; B, Supplementary Figure S6; C, Supplementary Figure S8.

A

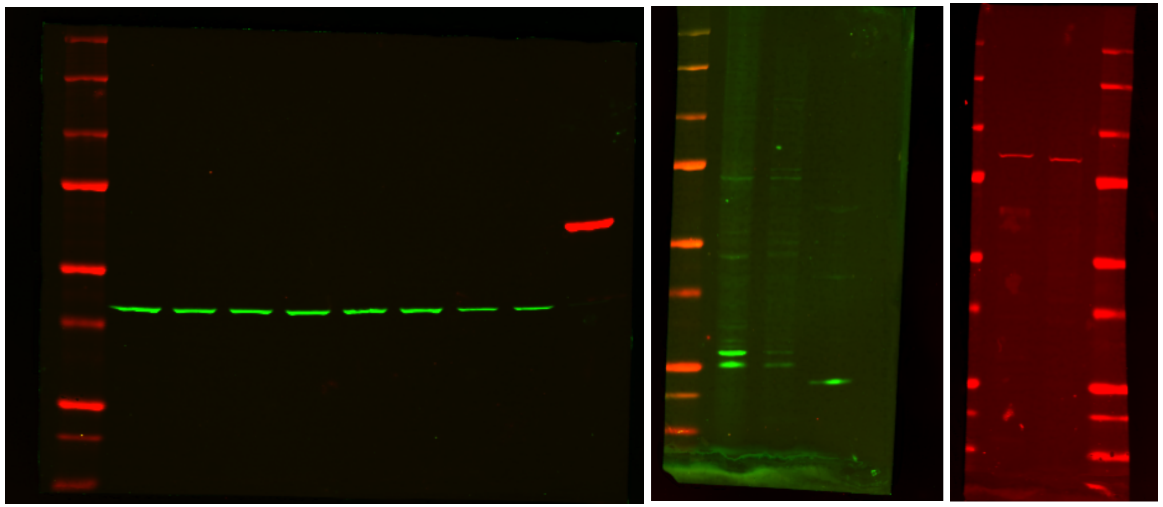

B

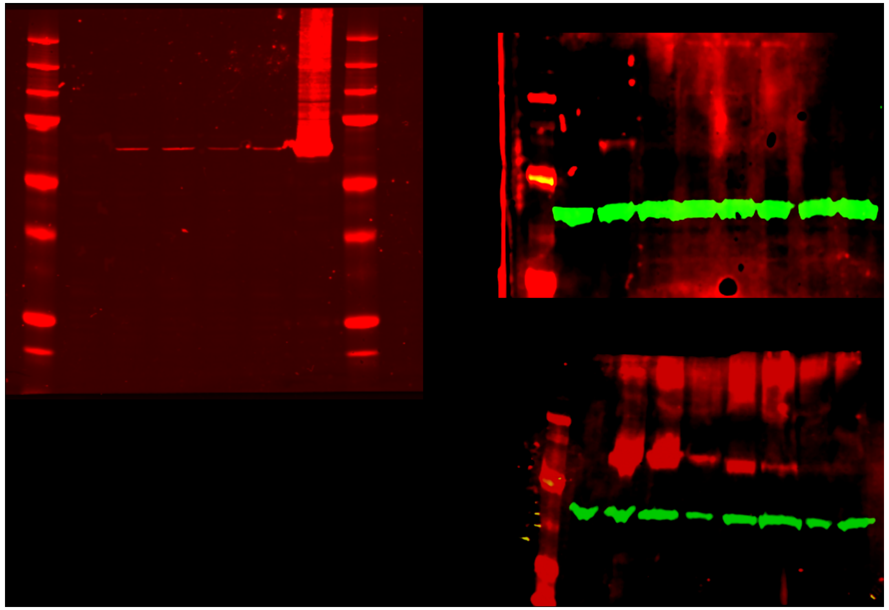

C

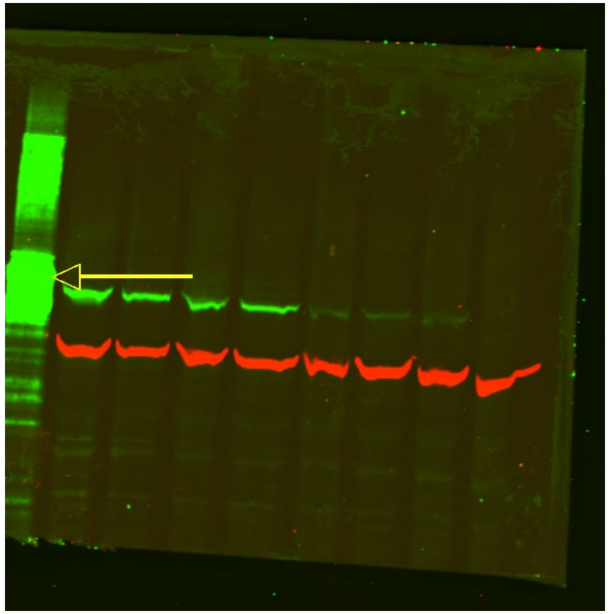

Supplement: Supplementary file 1 — Supplementary Material 1 [file 41598_2025_12926_MOESM1_ESM.pdf]
